# IL-1 receptor–associated kinase-3 acts as an immune checkpoint in myeloid cells to limit cancer immunotherapy

**DOI:** 10.1172/JCI161084

**Published:** 2023-04-03

**Authors:** Gürcan Tunalı, Marta Rúbies Bedós, Divya Nagarajan, Patrik Fridh, Irineos Papakyriacou, Yumeng Mao

**Affiliations:** Science for Life Laboratory, Department of Immunology, Genetics and Pathology, Uppsala University, Uppsala, Sweden.

**Keywords:** Immunology, Oncology, Cancer immunotherapy, Cellular immune response, Dendritic cells

## Abstract

Inflammatory mediators released by cancer cells promote the induction of immune suppression and tolerance in myeloid cells. IL-1 receptor–associated kinase-3 (IRAK3) is a pseudokinase that inhibits IL-1/TLR signaling, but its role in patients treated with immune checkpoint blockade (ICB) therapy remains unclear. Using RNA-Seq data from the IMvigor210 trial, we found that tumors with high *IRAK3* expressions showed enriched antiinflammatory pathways and worse clinical response to ICB therapy. Upon IRAK3 protein deletion with CRISPR/Cas9, primary human monocytes displayed altered global protein expression and phosphorylation in quantitative proteomics and released more proinflammatory cytokines in response to stimulation. Bone marrow–derived macrophages from an *IRAK3* CRISPR KO mouse model demonstrated a proinflammatory phenotype and enhanced sensitivity to TLR agonists compared with WT cells. *IRAK3* deficiency delayed the growth of carcinogen-induced and oncogene-driven murine cancer cells and induced enhanced activation in myeloid cells and T cells. Upon ICB treatment, *IRAK3-*KO mice showed enrichment of TCF1^+^PD-1^+^ stem-like memory CD8^+^ T cells and resulted in superior growth inhibition of immunologically cold tumors in vivo. Altogether, our study demonstrated what we believe to be a novel cancer-driven immune tolerance program controlled by IRAK3 in humans and mice and proposed its suitability as an immunotherapy target.

## Graphical abstract

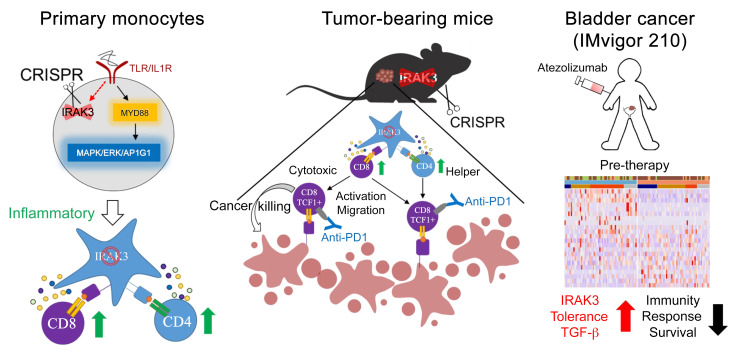

## Introduction

Tumor elimination by immune checkpoint blockade (ICB) therapy against programmed cell death protein 1 (PD-1) or its ligand PD-L1 has generated remarkable clinical benefits in patients with cancer (1). However, cancer cells can develop resistance to the therapy through the loss of immune recognition machinery or upregulation of immune inhibitory pathways (2). In addition, tumor-derived factors recruit a range of immunosuppressive myeloid cells and prevent efficient immune responses (3, 4). Functionally, suppressive myeloid cells dampen the response to ICB therapy through multiple mechanisms, including direct inhibition of antitumor immunity, prevention of immune cell infiltration, and maintenance of abnormal vasculature in the tumor microenvironment (5). It is widely accepted that cancer-driven chronic inflammation leads to insufficient activation of myeloid cell subsets and supports the expansion of myeloid cells with suppressive functions (4).

Myeloid cells are a pivotal line of immune defense during viral and bacterial infections. Pathogen-derived elements can activate myeloid cells through the toll-like receptors (TLRs) or IL-1 pathway. Upon activation, TLRs trigger assembly of the myddosome complex, which leads to the dissociation of phosphorylated IL-1 receptor–associated kinase-1 (IRAK1) and activation of multiple pathways such as NFκB, MAPK/ERK or AKT (6). As opposed to other IRAK family members, IRAK3 negatively regulates the TLR pathway in mice (7) by stabilizing the myddosome complex through its death domain (8). Mice lacking IRAK3 show greater sensitivity to bacterial infection and slower growth of implanted tumors in vivo (9–11). The intrinsic role of IRAK3 in cancer cells has also been proposed, where IRAK3 sustains the growth of colon cancer cells through the stabilization of STAT3 (10). In humans, published results suggest that cancer-derived factors could constrain the activation of human myeloid cells by enhancing IRAK3 expression (12). However, it remains unclear how IRAK3 contributes to immune tolerance in human myeloid cells and regulates response to immunotherapy in patients.

Here, we have thoroughly investigated the mechanistic importance of IRAK3 using CRISPR/Cas9-mediated genome editing in primary human monocytes and in immunocompetent mice. Our results show that IRAK3 is a key regulator for the inflammatory status of myeloid cells. Genetic deletion of *IRAK3* in immunocompetent mice enables efficient tumor growth inhibition and synergizes with ICB treatment. Our preclinical observations are confirmed in a patient data set with urothelial cancers, where the expression of *IRAK3* mRNA in pretreatment tumor tissues is associated with the inflammatory signatures and the treatment outcome of ICB therapy.

## Results

### IRAK3 expression predicts response to ICB therapy in patients with urothelial cancer.

**Figure 1:**
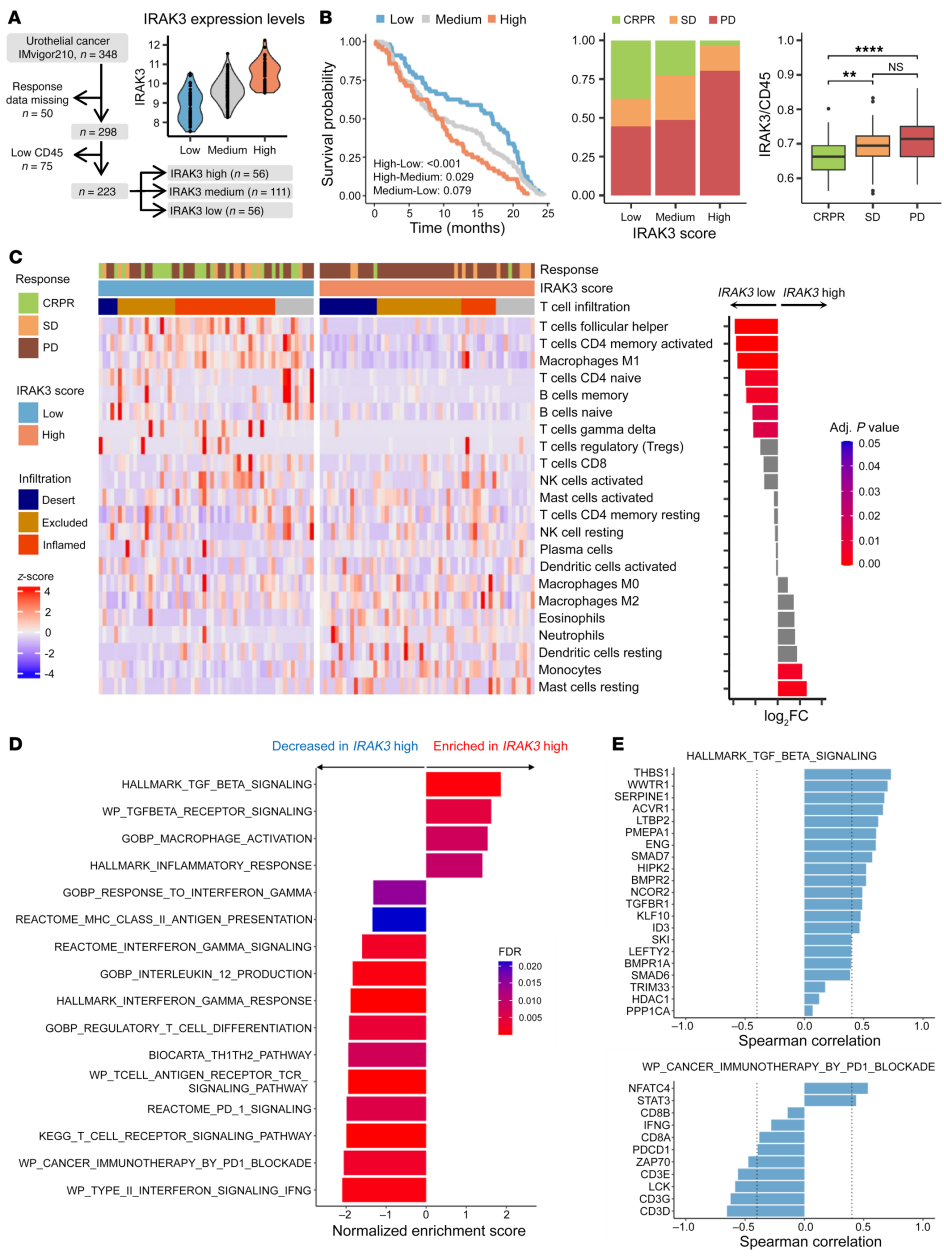
Pretherapy *IRAK3* level predicts response to ICB therapy. (**A**) RNA sequencing results from pretherapy tumor samples of the IMvigor210 trial were analyzed and patients with missing response data (n=50) or low CD45 expression (n=75) were excluded. The remaining patients were stratified according to IRAK3 expression levels. (**B**) Survival of patients expressing high (*n* = 56), medium (*n* = 111) or low (*n* = 56) *IRAK3* was compared using a Kaplan-Meier curve and fitted with a Cox proportional hazards regression model. Clinical response and *IRAK3* expression levels were compared using an ANOVA test with Tukey’s correction. **P < 0.01, ****P < 0.0001. (**C**) Immune deconvolution analysis was performed using CIBERSORTx and immune cell subsets in *IRAK3* high or *IRAK3* low patients were shown. A linear model was fitted to determine the differences in immune cell abundance between the 2 groups. (**D**) GSEA on differentially expressed pathways comparing *IRAK3* high and low patients. (**E**) Spearman correlation of *IRAK3* scores and differentially expressed genes in the TGFB signaling pathway or the activation of the adaptive immunity (adjusted *P* value < 0.001).

Although IRAK3 is reportedly a negative regulator for myeloid cells in mouse models, its role in the context of immunotherapy has yet to be elucidated. We tested the clinical relevance of *IRAK3* using RNA sequencing data from patients with locally advanced or with metastatic urothelial cancer treated with atezolizumab, an approved ICB therapy, in the IMvigor210 clinical trial (13). Because *IRAK3* is predominantly expressed by myeloid cells, we found strong correlations between *IRAK3* and immune cell markers including *CD45*, *CD11b*, and *CD11c* in pretherapy tumor samples (Supplemental Figure 1, A and B; supplemental material available online with this article; https://doi.org/10.1172/JCI161084DS1). In order to examine the distribution of *IRAK3* mRNA in the bladder cancer tumor microenvironment, we employed a published single-cell RNA-Seq data set (14). Our analysis revealed that *IRAK3* mRNA was absent in cancer cells and nonmyeloid immune cells but was found in fibroblasts and endothelial cells (Supplemental Figure 1C). To test whether *IRAK3*-expressing nonimmune cells could contribute to the clinical response to ICB therapy, we performed an analysis of the *CD45* low patient subset (*n* = 75) in the IMvigor210 cohort. As shown in Supplemental Figure 2A, *IRAK3* expression did not correlate to ICB response in these patients. Therefore, patients with low *CD45* expression (*n* = 75) or missing response data (*n* = 50) were excluded from the subsequent analyses (Figure 1A). *IRAK3* scores were generated by calculating ratios between *IRAK3* and *CD45*. Next, patients were stratified according to the pretherapy *IRAK3* scores (Figure 1A) and the response to treatment was compared. As shown in Figure 1B, patients with low (bottom 25%, *n* = 56) or medium (50%, *n* = 111) *IRAK3* scores showed improved survival after PD-L1 blockade therapy compared with patients with high *IRAK3* (top 25%, *n* = 56, *P* < 0.001 and *P* = 0.029, respectively). Of note, neither *CD11b* nor *CD45* expression alone predicted patient survival after therapy (Supplemental Figure 2B). Moreover, patients who experienced complete or partial responses showed significantly lower expression of *IRAK3* prior to therapy compared with patients with stable or progressive disease (Figure 1B). To investigate the prognostic value of *IRAK3*, we extracted RNA-Seq results of a subset of patients with advanced or metastatic urothelial cancer (*n* = 132) from The Cancer Genome Atlas (TCGA) (15). Notably, *IRAK3* score was not associated with the clinical outcome of these patients (Supplemental Figure 2C).

In order to reveal the intratumoral immune landscape in *IRAK3*-high and -low patients, we conducted immune deconvolution analysis using CIBERSORTx (16). Tumors with low *IRAK3* scores showed a significant enrichment of proinflammatory immune cell subsets, such as M1-like macrophages, T follicular helper cells, activated CD4^+^ memory T cells, memory B cells, whereas monocytes and mast cells were more abundant in *IRAK3*-high patients (Figure 1C). Next, we sought to investigate the transcriptional network associated to *IRAK3* in patients in the IMvigor210 trial. We identified immune regulatory genes that were either significantly upregulated (*TGFB1I1*, *MMP10*, *ITGAV*, *VNN1*, and *ALDOB*) or downregulated (*CXCR5*, *KRT1*, *KRT2*, *PAX5*, *LILRA4*, *CD79B*, and *FCER2*) in patients with high *IRAK3* scores (Supplemental Figure 2D). Pathway analysis on differentially expressed genes demonstrated amplified TGFB signaling, altered macrophage activation, and inflammatory response in patients with high *IRAK3* scores. In contrast, dampened adaptive immunity, reduced antigen presentation, and weakened response to immune-stimulatory cytokines were observed in the same patient group (Figure 1D). In accordance, genes involved in TGFB signaling positively correlated with *IRAK3* expression, while genes involved in activation of adaptive immunity, such as *LCK*, *ZAP70*, and *CD3*, demonstrated reverse correlations (Figure 1E).

### Genetic deletion of IRAK3 alters the translational profile in primary human monocytes.

**Figure 2:**
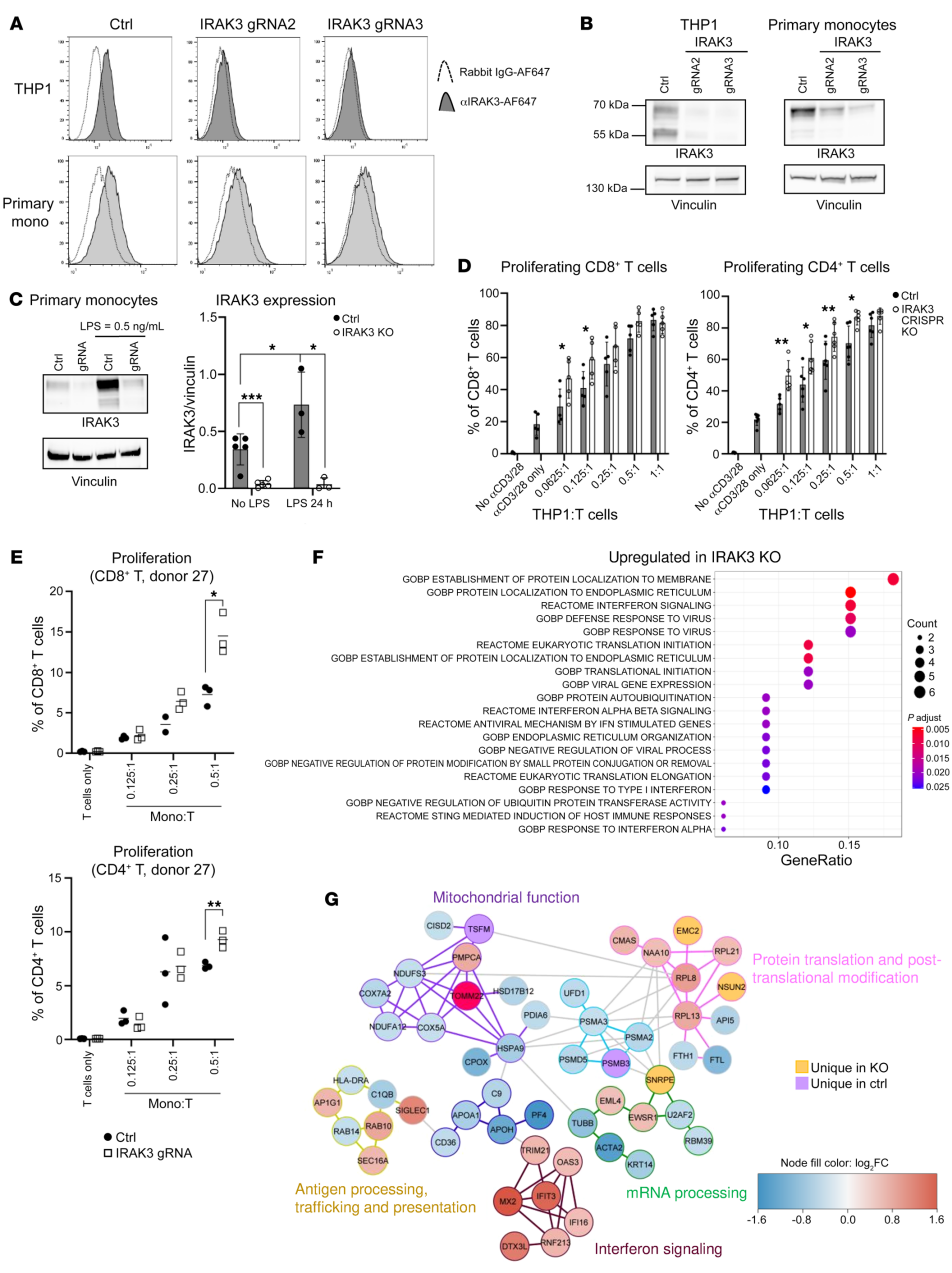
Genetic deletion of *IRAK3* modulates primary human monocytes. Human primary monocytes isolated from healthy blood donors or the human monocytic THP1 cell line were transfected with recombinant Cas9 and RNP complexes targeting the human *IRAK3* gene. Deletion of the IRAK3 protein in human monocytic THP1 cells and primary monocytes was confirmed using (**A**) flow cytometry and (**B**) Western blotting. Cells transfected with Cas9 and the tracrRNA were used as controls. Representative histogram and blots from 3 experiments. (**C**) Primary human monocytes transfected with RNP complex with or without the *IRAK3*-targeting gRNA were cultured in 100 ng/mL rhGM-CSF and activated with LPS at 0.5 ng/mL. The resulted expression of the IRAK3 protein was shown in at least 3 donors (mean ± SD, unpaired *t* test, *P < 0.05, ***P < 0.001). (**D**) Control or IRAK3 KO THP1 cells were cocultured with primary human T cells from at least 5 donors in presence of micro-beads coated with αCD3/CD28 antibodies. The proliferation of CD4^+^ or CD8^+^ T cells was assessed using flow cytometry after 4 days (mean ± SD, unpaired *t* tests. *P < 0.05, **P < 0.01). (**E**) Primary human monocytes isolated from fresh buffy coats were transfected with RNP complex with or without the *IRAK3*-targeting gRNA. Cells were harvested after 4 days and cocultured with allogeneic CD3^+^ primary human T cells at indicated ratios. The proliferation of T cells was assessed after 5 days by flow cytometry, data were analyzed using unpaired *t* tests. *P < 0.05, **P < 0.01. Representative donor of 3 biological repeats. (**F**) Global protein expression in control or IRAK3-KO primary human monocytes from 4 donors after treatment with 1 μg/mL LPS for 45 minutes were determined using quantitative proteomics analysis and upregulated pathways in KO cells were shown. (**G**) Proteins were clustered by community analysis and grouped based on their functions. Expressions of individual proteins in IRAK3-KO versus control were shown by colors. Red, upregulated in KO; blue, downregulated in KO; purple, only detected in control; yellow, only detected in KO. The outline of the proteins and the community they belong to were annotated according to the biological functions described in the graph. Statistical tests were performed using paired student’s *t* tests and corrected by the False Discovery Rate.

Because *IRAK3* mRNA expression is associated with patient response to immunotherapy in our analysis, we investigated the putative underlying mechanisms of IRAK3 in human primary monocytes using CRISPR/Cas9 genome editing. To avoid immune activation due to viral transduction, we cotransfected a recombinant Cas9 protein with guide RNAs (gRNAs) targeting the human *IRAK3* gene into human THP1 monocytic cell line and identified gRNAs that were efficient in reducing IRAK3 protein expression (Supplemental Figure 3A). The selected gRNAs were used to achieve protein deletion in a protocol optimized for primary human monocytes (Figure 2, A and B; see complete unedited blots in the supplemental material). As expected, expression of the IRAK3 protein in primary monocytes was elevated after treatment with LPS, which was robustly suppressed using the CRISPR/Cas9 protocol across donors (Figure 2C). Functionally, IRAK3-deficient THP1 cells induced significantly higher proliferation of CD4^+^ and CD8^+^ T cells when cocultured with primary human T cells in the presence of αCD3/CD28 beads (Figure 2D and Supplemental Figure 3B). When primary human T cells were cocultured with monocytes in a mixed lymphocyte reaction, we observed increased T cell proliferation primed by IRAK3-KO monocytes (Figure 2E).

In order to map the translational landscape regulated by IRAK3, we conducted simultaneous proteomics and phospho-proteomics analysis in IRAK3 KO and control monocytes after a short stimulation with LPS. We identified AP1G1 to be among the most upregulated proteins upon IRAK3 protein deletion in primary monocytes, which is known to be involved in gene transcription downstream of the MAPK/ERK pathway (Supplemental Figure 3C). Further pathway enrichment analysis demonstrated that IRAK3 protein deletion potentiated response to interferons and viruses in human primary monocytes (Figure 2F). When grouped according to biological functions, we observed that proteins related to interferon signaling, including MX2, IFIT3, IFI16, DTX3L, OAS3, RNF213, and TRIM21, were upregulated in primary human monocytes as a result of IRAK3 protein deletion (Figure 2G).

### TLR-induced signaling network and cytokine release are governed by IRAK3 in primary human monocytes.

**Figure 3:**
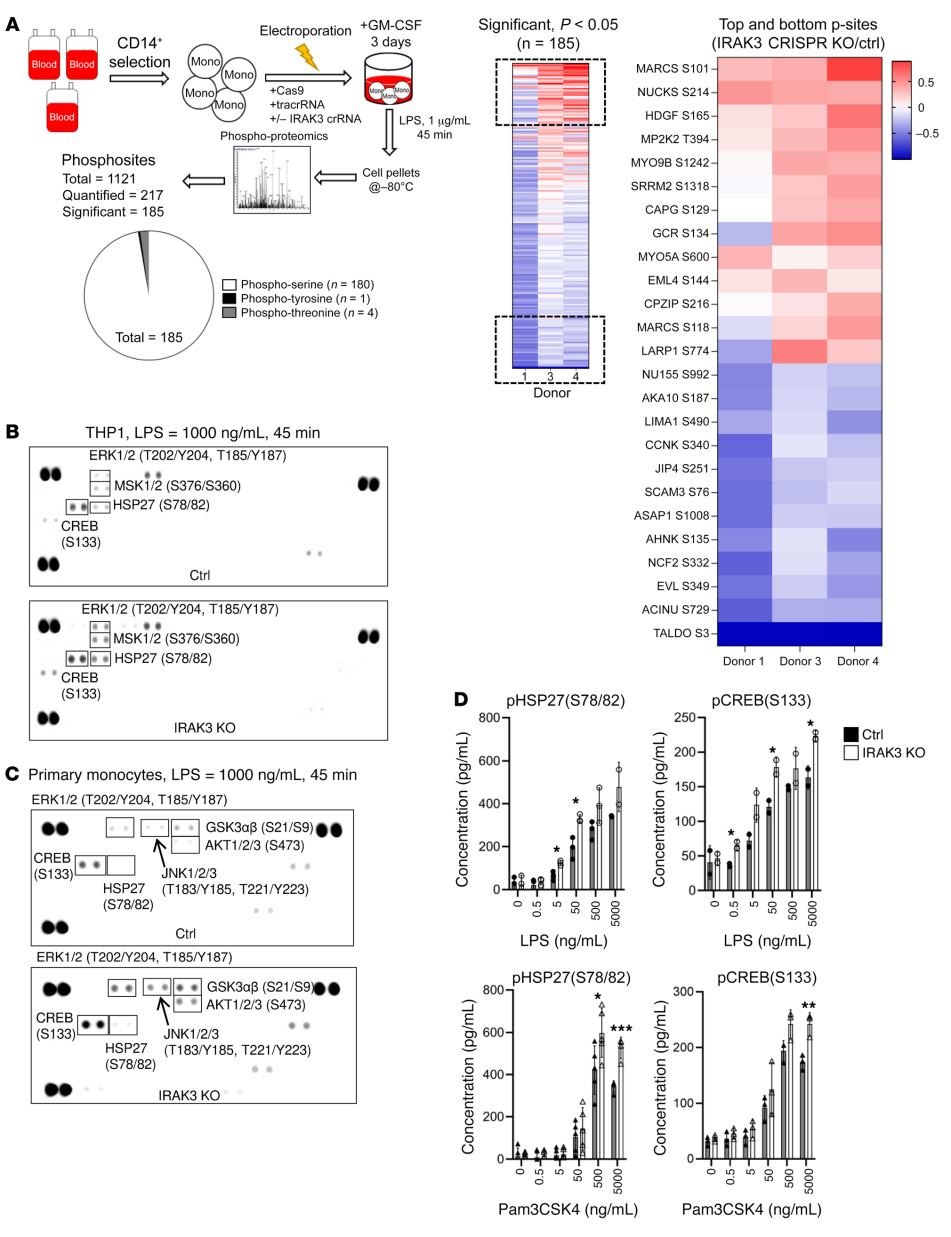
IRAK3 deletion amplifies signaling through TLR in primary human monocytes. (**A**) Experimental flow for the phospho-proteomics analysis in control or IRAK3-KO primary monocytes isolated from 3 donors. The most significantly changed phosphosites after IRAK3 deletion in monocytes were shown. Detection of phosphorylated proteins using a phospho-kinase array in control or IRAK3 KO (**B**) THP1 cells or (**C**) primary human monocytes, in response to 1 μg/mL LPS treatment for 45 minutes. Representative membranes from 2 repeats was shown. (**D**) Individual detection of pCREB(S133) or pHSP27(S78/82) by ELISA in control or IRAK3-KO THP1 cells in response to increasing concentrations of LPS or Pam3CSK4 for 45 minutes. Data were summarized from 2–5 biological repeats and shown as mean ± SD. Statistical tests were performed using unpaired *t* tests. *P < 0.05, **P < 0.01, ***P < 0.001.

Global phospho-proteomics analysis on primary monocytes identified significant changes on 185 phosphosites as a result of IRAK3 protein deletion (Figure 3A). Several altered phosphosites, such as JIP4, HDGF, and MYO9B, in IRAK3 deficient cells were reported to regulate the TLR pathway. In particular, phosphorylation of MAPK kinase 2 (MP2K2 T394) was among the most upregulated phosphorylated peptides. Because the MAPK/ERK pathway plays a key role in regulating LPS-induced immune activation, we validated the phospho-proteomics data using a human phospho-kinase array. As shown in Figure 3, B and C, deletion of IRAK3 protein enhanced the phosphorylation of ERK1/2, JNK, AKT, CREB, and HSP27, all of which could be regulated by the MAPK pathway. These results were confirmed across different LPS concentrations, thereby excluding that this effect was due to TLR-agonist concentrations (Figure 3D). Furthermore, upon activation through TLR1/2 with Pam3CSK4, enhanced phosphorylation of CREB (S133) and HSP27 (S78/82) was observed in IRAK3-KO THP1 cells (Figure 3D).

**Figure 4:**
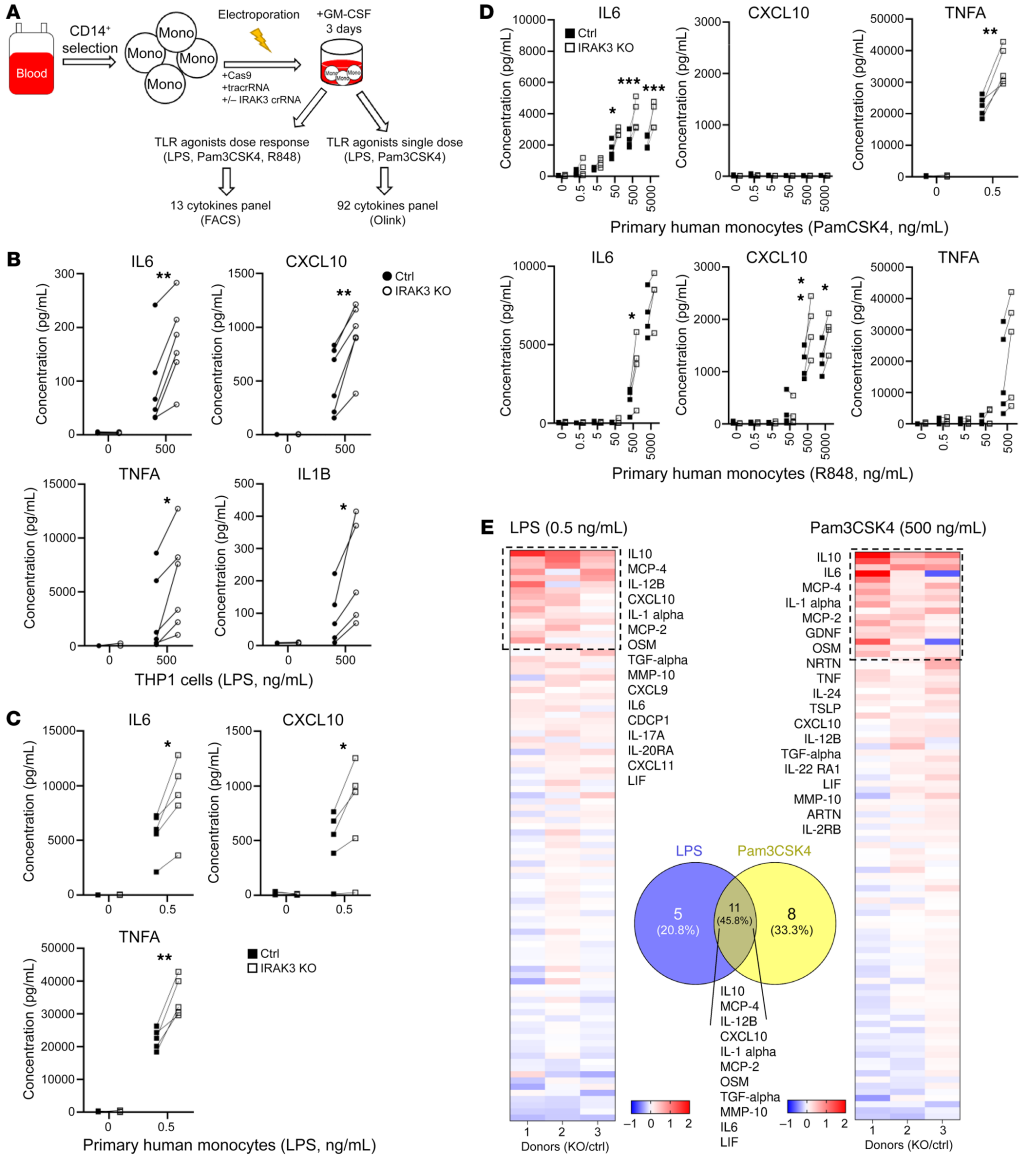
Cytokine release is controlled by IRAK3 in human primary monocytes. (**A**) Experimental flow for the cytokine analysis in control or IRAK3-KO primary monocytes or THP1 cells. (**B**) Release of cytokines in control or IRAK3-KO THP1 cells in response to 500 ng/mL LPS treatment for 5 hours was shown. Cytokines released from control or IRAK3-KO primary monocytes treated with (**C**) 0.5 ng/mL LPS or (**D**) increasing concentrations of Pam3CSK4 or R848 after 5 hours were summarized. Number of experiments shown as individual dots. (**E**) A panel of 92 soluble factors released by control or IRAK3-KO monocytes in response to activation was assessed using the Olink technology. Ratio changes in KO/WT were presented in the heatmaps. Statistical tests for all figures were performed using unpaired *t* tests. *P < 0.05, **P < 0.01, ***P < 0.001.

In order to investigate whether the enhanced intracellular signaling upon IRAK3 protein deletion alters sensitivity to TLR stimulation in human myeloid cells, we established an in vitro assay to quantify soluble factors released by primary monocytes in response to TLR activation (Figure 4A). We observed that IRAK3 KO THP1 cells and primary monocytes released significantly higher IL-6, TNFA, CXCL10, and IL-1B in response to LPS treatment (Figure 4, B and C). Similarly, treatment with TLR1/2 agonist (Pam3CSK4) or TLR7/8 agonist (R848) resulted in increased production of IL6 and TNFA from primary human monocytes upon IRAK3 protein deletion (Figure 4D). Further analysis of 92 inflammatory mediators confirmed the results on CXCL10, IL-6, and TNF, and revealed additional factors regulated by IRAK3, including IL12B and IL6 family member proteins OSM and LIF, MMP10, IL-10, and MCP2/4 (Figure 4E).

To prove that MAPK/ERK signaling is required for cytokine release in IRAK3-KO cells, we pretreated IRAK3-KO THP1 cells with pharmacological inhibitors, followed by stimulation with LPS. Our results showed that inhibition of MAPK or ERK abrogated the production of TNFA from IRAK3-KO THP1 cells. MAPKi, but not ERKi, showed a decrease in CXCL10 release (Supplemental Figure 3D). Of note, IRAK3 protein deletion did not affect PD-L1 expression induced by LPS on THP1 cells (Supplemental Figure 3E), nor did it have an impact on the release of CXCL10 and IFNB, or expression of PD-L1 and HLA-ABC after treatment with a STING agonist, i.e., ADU-S100 (Supplemental Figure 3, F and G).

### Germline deletion of IRAK3 by CRISPR/Cas9 enhances the proinflammatory property of macrophages.

**Figure 5:**
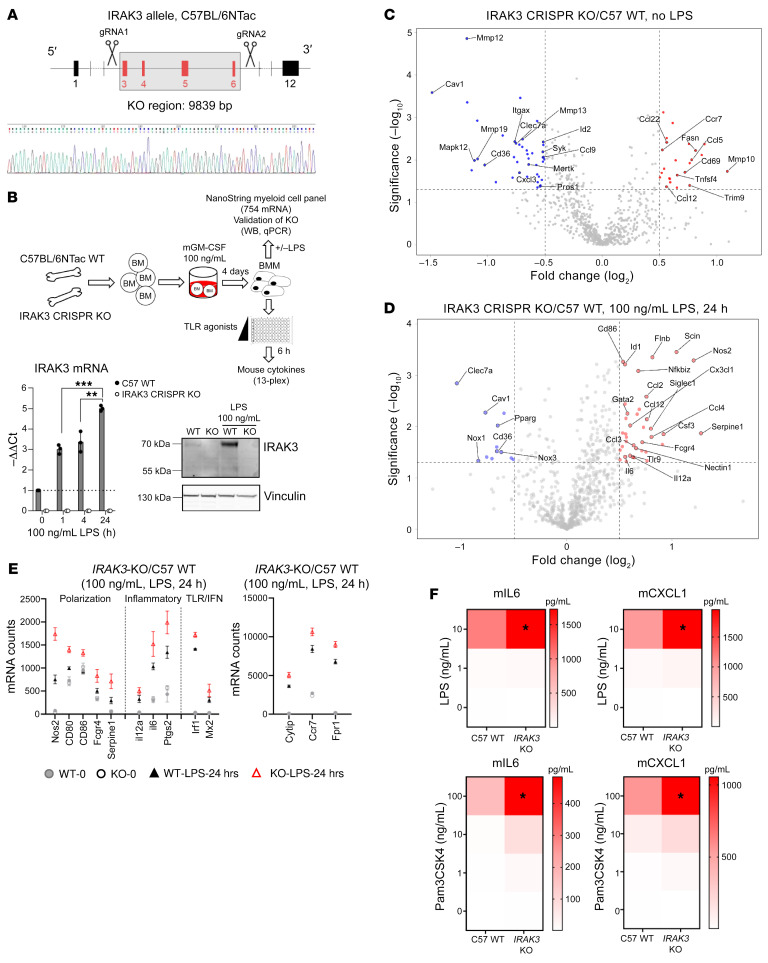
Deletion of *IRAK3* by CRISPR/Cas9 in mice enhances myeloid cell function. (**A**) Design of the *IRAK3 CRISPR–*KO mice and confirmation with DNA sequencing. Bone marrow cells were isolated from age-matched female C57BL/6NTac *WT* or *IRAK3 CRISPR–*KO mice and differentiated in the presence of 100 ng/mL rmGM-CSF for 4 days to macrophages (BMM). (**B**) Expression of *IRAK3* mRNA (*n* = 3) and protein in response to LPS treatment was measured using qPCR (mean ± SD, unpaired *t* tests, **P < 0.01, ***P < 0.001) or Western blotting (representative blot from 3 biological repeats). A panel of 754 myeloid cell related genes were measured when *WT* or *IRAK3-*KO BMM cells (*n* = 3 of each) were (**C**) cultured in media or (**D**) activated with 100 ng/mL LPS for 24 hours. Data was plotted using log_2_ fold changes and log_10_ *P* values, unpaired student *t* tests. (**E**) Changes in expression of selected genes were shown, mean ± SD. (**F**) BMM cells from at least 4 *WT* or *IRAK3-*KO mice were seeded in a 96-well plate and activated with increasing concentrations of LPS or Pam3CSK4 and concentrations of soluble mIL6 or mCXCL1 in culture supernatants were shown after 5 hours. Statistical tests were performed using unpaired *t* tests. *P < 0.05.

To dissect the role of IRAK3 in vivo, we created a germline *IRAK3-KO* mouse model using CRISPR/Cas9 on the C57BL/6NTac background (Figure 5A). In brief, gRNAs were designed to target 4 exons on the mouse *IRAK3* gene. F1 mice with the largest deletion region (9839 bp) were selected. Homozygous KO mice were validated using DNA sequencing and PCR genotyping (Figure 5A and Supplemental Figure 4A). Similar to human primary monocytes, LPS treatment induced rapid transcription and translation of IRAK3 in murine bone marrow–derived macrophages (BMM), both of which were absent in BMM from CRISPR KO mice (Figure 5B; see complete unedited blots in the supplemental material). Next, we sought to evaluate the effect of *IRAK3* gene deletion on macrophage activation. Expression of 754 myeloid cell–related genes was quantified in control or *IRAK3-KO* BMM at the baseline or activated with LPS using a Nanostring panel. In nontreated BMM, *IRAK3* deletion led to the downregulation of genes associated with immunosuppressive myeloid cells, such as *MERTK* and *CD36* and upregulation of cell migratory genes, including *CCR7* and *CCL12* (Figure 5C). As expected, LPS induced substantial changes in gene expression in BMM (Supplemental Figure 4B), and *IRAK3-*KO BMM demonstrated the upregulation of genes associated with proinflammatory responses such as *NOS2*, *IL-12A*, *CD80*, *CD86*, *IL-6*, *MX2*, and *IRF1* (Figure 5, D and E). In accordance, the release of IL-6 and CXCL1 to culture supernatants was significantly increased in *IRAK3-*KO BMM after TLR stimulation (Figure 5F). Release of IL-12p40, TNFA, CCL17, and CCL22 showed increased trends in *IRAK3-*KO BMM (Supplemental Figure 4C).

### Concurrent activation of innate and adaptive immunity constrains tumor growth in vivo.

**Figure 6:**
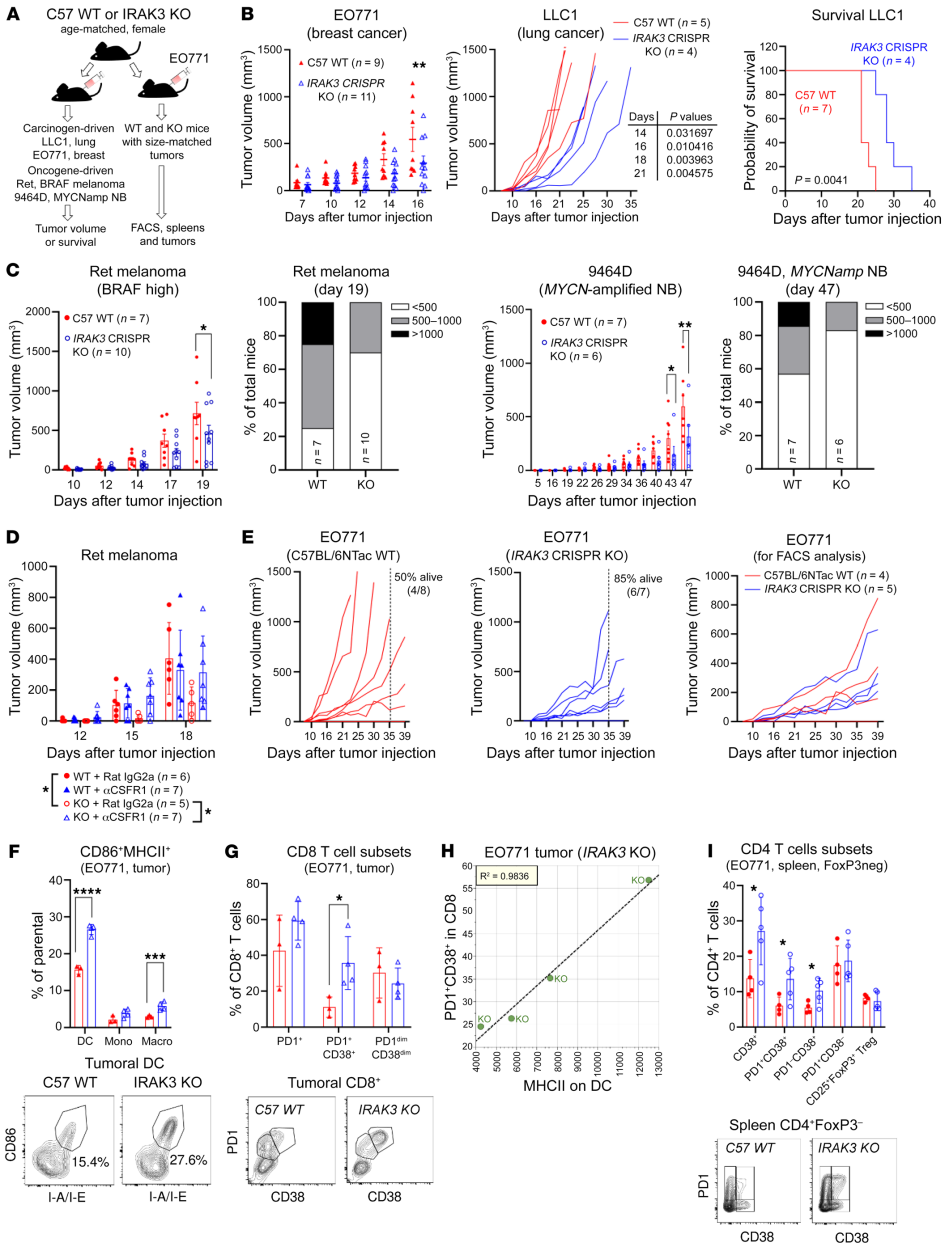
Concurrent changes in innate and adaptive immunity are regulated by *IRAK3* in vivo. (**A**) Experimental design to compare tumor growth and immunological changes in *WT* and *IRAK3-*KO mice. (**B**) Syngeneic breast cancer cell line EO771 (400,000 cells per mouse, WT = 9, KO = 11, mean ± SEM) or lung cancer cell line LLC1 (200,000 cells per mouse, WT = 5, KO = 4) were injected subcutaneously in *WT* or *IRAK3-*KO mice. Tumor growth and survival of the animals were shown in a representative experiment of 2 repeats. (**C**) Oncogene-driven murine Ret melanoma cells (50,000 cells per mouse, WT = 7, KO = 10, mean ± SEM) or *MYCN*-amplified 9464D neuroblastoma cells (600,000 cells per mouse, WT = 7, KO = 6, mean ± SEM) were injected s.c. in *WT* or *IRAK3-*KO mice. Tumor growth of individual mouse and tumor size distribution at the study endpoints were shown. Representative experiment of 3 repeats. (**D**) Rat IgG2a isotype or a CSF-1R depleting antibody was i.p. injected into *WT* or *IRAK3-*KO mice in 100 μL PBS 3 days prior to implantation of Ret melanoma cancer cells (50,000 cells per mouse s.c.). Tumor volumes of individual mice were shown as mean ± SEM. The effect of CSF-1R antibody (clone AFS98) on tumor-bearing *IRAK3-*KO mice was observed in 3 individual experiments. (**E**) To study the immunological changes in size-matched tumors, EO771 cells (200,000 per mouse) were injected s.c. in *WT* or *IRAK3-*KO mice. Mice bearing comparable sizes of tumors (WT = 3 and KO = 4) on day 39 after injection were selected for FACS analysis. (**F** and **G**) Immunological changes and (**H**) correlation between PD-1^+^CD38^+^ cytotoxic T cells and MHCII on dendritic cells in tumor tissues were shown in tumor-bearing KO mice (*n* = 4). (**I**) Changes of CD4^+^ T cell subsets in spleens of the tumor-bearing mice were shown. All studies used age-matched female WT or *IRAK3-*KO mice. Statistical tests were performed using unpaired *t* tests. *P < 0.05, **P < 0.01, ***P < 0.001, ****P < 0.0001.

To test whether IRAK3 deficiency has an effect on tumor growth, syngeneic murine cancer cell lines were injected subcutaneously in age-matched WT or *IRAK3 CRISPR-*KO mice (Figure 6A). Murine breast cancer cell line EO771 and lung cancer cell line LLC1 showed significantly delayed growth in *IRAK3-*KO mice, leading to prolonged survival of the LLC1 tumor–bearing mice (Figure 6B). To model tumors with low mutational burden, we employed oncogene-driven cell lines that were derived from spontaneous tumors in transgenic mice, namely the Ret melanoma cell line (17) and the *MYCN*-amplified neuroblastoma cell line 9464D. As shown in Figure 6C, Ret melanoma demonstrated an aggressive growth behavior, while 9464D tumors had a long onset period. Of note, removal of *IRAK3* in mice resulted in significantly delayed tumor growth in both models and substantially increased the proportion of small tumors at the study endpoint. In 9464D tumors, we identified significantly enhanced infiltration of TNFA^+^IFNG^+^ and TNFA^+^PD-1^–^ T cells in *IRAK3*-deficient mice (Supplemental Figure 5A). Because *IRAK3* deletion was in the germline in our model, we tested the lineage-specific effects on tumor growth using a myeloid cell depletion antibody against murine CSF-1R. Depletion of myeloid cells reverted the tumor growth delay in Ret melanoma-bearing *IRAK3-*KO mice but had no impact in the WT counterparts (Figure 6D).

In order to assess tumor-driven immunological changes using flow cytometry, we selected EO771 tumors that showed comparable growth rates in WT and IRAK3-KO mice (Figure 6E). By using a multivariant analysis, we identified several immune populations that were directly associated with tumor volumes (Supplemental Figure 5B). Among the cell populations that were not impacted by the tumor volume, we observed significant enrichment of activated dendritic cells and macrophages (CD86^+^MHCII^+^) in tumors from *IRAK3-*KO mice (Figure 6F and Supplemental Figure 5, B and C). Moreover, a subset of CD8^+^ T cells that coexpress PD-1 and CD38 was only found in tumor tissues and was significantly upregulated as a result of IRAK3 deletion (Figure 6G). These two cell populations showed a strong correlation in *IRAK3-*KO tumor–bearing mice (Figure 6H and Supplemental Figure 5D). In the spleens of tumor-bearing mice, we observed significantly elevated CD38^+^ cells in FoxP3 negative CD4^+^ T cells (Figure 6I). In contrast, immunological changes of splenic myeloid cells and cytotoxic T cells were not statistically significant (Supplemental Figure 5E).

### Deletion of IRAK3 triggers local and systemic remodelling of antitumor immunity in response to ICB therapy.

**Figure 7:**
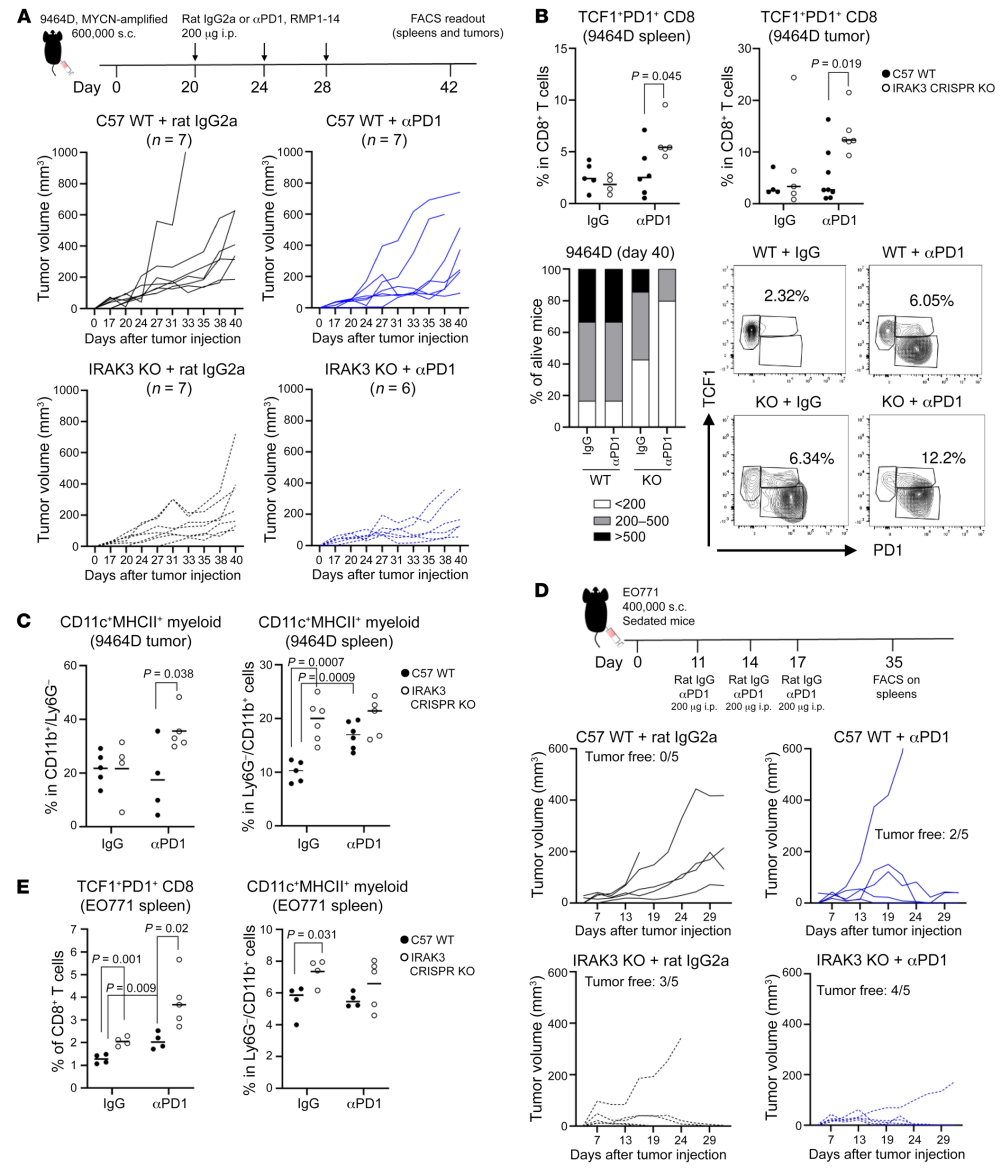
ICB treatment induces distinct immunological changes in *IRAK3 KO* mice. (**A**) *MYCN*-amplified 9464D murine neuroblastoma cells were injected s.c. in *WT* or *IRAK3-*KO mice. Group sizes were indicated in the figures. When tumors were measureable in 80% of the mice, 200 μg anti-PD-1 antibody (clone RMP1-14) or a Rat IgG2a isotype control antibody was infused i.p. in 100 μL PBS per mouse on days 20, 24, and 28 in *WT* or *IRAK3-*KO mice. Tumor growth of individual mice and tumor volume distribution at the study endpoint were shown. Frequencies of the (**B**) TCF1^+^PD-1^+^ stem-like CD8^+^ T cells or (**C**) CD11c^+^MHCII^+^ myeloid cells were shown in treatment groups in the tumors or in the spleens. (**D** and **E**) PD-1 blockade–sensitive EO771 cancer cells (400,000 cells per mouse) were injected s.c. in *WT* and *IRAK3-*KO mice, followed by treatment with the PD-1 blocking antibody or the Rat IgG2a isotype control antibody on days 11, 14, and 17. Tumor volumes and immunological changes in the spleens at the study endpoint were shown. All studies used age-matched female *WT* or *IRAK3-*KO mice. Statistical tests were performed using unpaired *t* tests and *P* values were shown on the figures.

Next, we sought to investigate the antitumor efficacy in response to ICB therapy in mouse models. We selected the *MYCN*-driven mouse neuroblastoma cell line 9464D because it has low mutational burden and was not responsive to ICB treatment (18) (Figure 7A). Consistent with earlier results, germline deletion of *IRAK3* resulted in delayed tumor growth in mice treated with the isotype control antibody (Figure 7A). A total of 80% of *IRAK3-*KO mice receiving a PD-1 blocking antibody showed small tumors (< 200 mm^3^) at day 40 after tumor implantation, compared with 16% of anti-PD-1–treated WT mice (Figure 7A). Next, we assessed immunological changes in tumors and spleens using flow cytometry. Our data showed a significant increase of the TCF1^+^PD-1^+^ stem-like CD8^+^ T cells in *IRAK3*-KO mice treated with the PD-1 blocking antibody, compared with anti-PD-1–treated WT counterparts (Figure 7B and Supplemental Figure 6A). The frequency of CD25^+^PD-1neg cells were significantly increased in FoxP3^–^ CD4^+^ T cells in this group (Supplemental Figure 6B). Moreover, activation of dendritic cells and frequency of CD38^+^ cytotoxic T cells in tumor tissues were significantly elevated by the PD-1 blockade in *IRAK3*-KO mice (Figure 7C and Supplemental Figure 6B). In the spleens of these mice, activation of myeloid cells was driven by either *IRAK3* deletion or the PD-1 blockade alone (Figure 7C). In order to exclude that the immunological changes were due to tumor sizes, we employed the EO771 breast cancer model that was sensitive to the PD-1 blockade. As shown in Figure 7D, 40% of anti-PD-1 treated control mice or 60% of the isotype-treated *IRAK3*-KO mice were tumor free. Of note, 4 out of 5 (80%) *IRAK3-*KO mice showed no detectable tumors after anti-PD-1 therapy. When comparing immunological changes in the spleens, anti-PD-1 treatment or *IRAK3* deletion resulted in significantly increased TCF1^+^PD-1^+^ stem-like CD8^+^ T cells. Of note, this population in *IRAK3-*KO mice receiving the PD-1 blocking antibody was significantly higher compared with other groups (Figure 7E). Similar to spleens from the 9464D-bearing model, *IRAK3* gene deletion enhanced the percentage of activated dendritic cells in the EO771-bearing mice, but PD-1 blockade did not further expand this population (Figure 7E). Immunological changes in the spleens of tumor-bearing mice were comparable between the 2 models but the baseline frequency of regulatory T cells (CD25^+^FoxP3^+^CD4^+^ T cells) was higher in the EO771 model (Supplemental Figure 6C).

## Discussion

A range of negative regulatory proteins against the TLR/IL-1 pathway tightly controls activation of innate immunity. The biological functions of these pathways have been studied by germline gene deletion in immunocompetent mice. Deficiency of SOCS1 (19), A20 (20), or TAM kinases (21) leads to lethal abnormality due to severe autoimmunity, indicating their broad regulatory functions in the immune system. In contrast, mice lacking two-thirds of the IRAK3 pseudokinase domain — i.e., a 1,200 bp gene deletion — are viable and normal in development (7), potentially due to its highly restricted function in myeloid cells (6, 21). To confirm that *IRAK3* deletion is not toxic to mice, we created a knockout mouse model using CRISPR/Cas9 technology, where a larger fragment (9893 bp) of the *IRAK3* gene is deleted. The homozygous KO mice show no signs of developmental or immune-related abnormality. Therefore, therapeutic targeting of IRAK3 has low risk in causing severe systemic autoimmunity.

The signaling pathways and effector functions of IRAK3 have been studied in mice (7) but are much less understood in human primary monocytes. We have combined proteomics, phospho-proteomics, and cytokine arrays to reveal key alterations when IRAK3 is deleted in primary human monocytes by CRISPR/Cas9. Our data show that IRAK3 governs cellular functions through the regulation of MAPK/ERK/AP1G1 axis and enhances the phosphorylation of key proteins such as CREB and HSP27. In accordance, inflammatory cytokines such as TNFA, CXCL10, IL-12B, and IL-6 family members are produced at higher levels in IRAK3-KO monocytes. We observed enhanced production of IL-10 upon IRAK3 protein deletion, which is a direct product of CREB activation (22). IL-10 has dual functions in regulating immunity and has been shown to amplify immunosuppressive functions in myeloid cells as well as to trigger potent antitumor immunity in patients with cancer (23). The precise function of IL-10 in this context remains to be elucidated.

Although the biology of IRAK3 has been reported in experimental models, there is scarce evidence to show its clinical relevance and therapeutic potential in cancer immunotherapy. In patients with melanoma treated with a CTLA4 blocking antibody, *IRAK3* mRNA in the blood is included in a 4-gene panel to predict patient outcome (24). We show here that the pretherapy *IRAK3* level in patients with advanced or metastatic urothelial cancer predicts response to ICB therapy in the IMvigor210 trial. Notably, *IRAK3* mRNA levels appear to be a biomarker of response to ICB therapy in patients with advanced or metastatic bladder cancers, because it is not associated to clinical outcome in a comparable patient subset from a TGCA cohort (15). However, further studies are needed to explore the prognostic value of *IRAK3* in other cancer types.

Our pathway analysis shows that *IRAK3*-high tumors coincide with enriched TGFB signaling. This is in line with the original study (13), where the authors identified and validated the TGFB pathway to be a resistant mechanism to ICB therapy. We have previously reported the role of TGFB in enabling immunosuppressive myeloid cells (25), which could be partially due to the induction of IRAK3 (9). Further, our findings in this study provide insights into immunosuppressive mechanisms in the tumor microenvironment, where IRAK3 serves as a key regulator for signal transduction, antigen presentation, inflammatory response, and response to ICB therapy.

In what we believe to be a novel *IRAK3 KO* mouse model created by CRISPR/Cas9, we demonstrated that *IRAK3* was a myeloid cell specific immune checkpoint. Using a range of carcinogen-induced or oncogene-driven murine tumor models, our data show that *IRAK3* deficiency resulted in enhanced activation of myeloid cells in tumor tissues, which could improve the migratory and antigen-presentation capacity of the cells (26). Depletion of myeloid cells in the germline KO model compromised tumor growth delay. Importantly, *IRAK3* deficient mice showed enhanced response to PD-1 blockade in an ICB-refractory *MYCN*-driven neuroblastoma tumor model. High-risk neuroblastoma is an aggressive form of childhood cancer known to resemble features of a ‘cold tumor’ and is unresponsive to ICB in preclinical models (27, 28). Here, we identified a concurrent increase of the TCF1^+^PD-1^+^ stem-like CD8^+^ T cells and highly activated myeloid cells in *IRAK3-*KO mice treated with the PD-1 blockade. Emerging evidence suggests that this subset of cytotoxic T cells plays a critical role in efficacious response after immunotherapy in mice (29, 30) and correlates to clinical response in patients (31). We propose that the IRAK3-regulated activation status of the innate immune cell types sustains the function of CD8^+^ T cell subsets. Mechanistic studies are warranted to determine the causal relationship between these 2 cell populations.

Because IRAK3 is a pseudokinase that lacks catalytic functions, it is considered an undruggable target for ATP–competitive small molecule drugs. We and others have shown that IRAK3 harbors a binding pocket accessible for chemical probes on the pseudokinase domain (32, 33). Selective chemical binders to the IRAK3 protein have been developed into proteolysis targeting chimera (PROTAC) molecules and mediate target-specific degradation in THP1 cells in vitro (33). However, it remains unclear whether these novel chemical compounds can mediate sustained target degradation and tumor growth inhibition in vivo. Because transcription and translation of *IRAK3* are strongly enhanced upon pathway activation, robust in vivo target engagement assays and pharmacodynamics biomarkers are required to guide dose-scheduling regimen in patients. Alternative targeting strategies against IRAK3 using chemical compounds include allosteric or protein-protein inhibitors that disrupt the binding of IRAK3 to other proteins in the myddosome complex. RNAi-based therapeutics can also be employed to limit translation of the IRAK3 protein, but this modality may carry off-target liability (34) or result in partial inhibition of protein synthesis. Although our study revealed clinical and mechanistic insights of IRAK3 as a drug target in synergy with ICB therapy, further hypotheses could be explored to uncover the link between IRAK3 and the microbiome, immunogenic cell death, and cancer metastasis.

## Methods

Specific information related to the reagents can be found in Supplemental Tables 1–3.

### Isolation of primary human monocytes.

Peripheral blood mononuclear cells (PBMC) were isolated from buffy coats using the Lymphoprep solution and the SepMate tubes (both from StemCell Technologies). Red blood cells were removed by incubating cells in red blood cell lysis buffer (Biolegend) for 10 minutes at room temperature. Primary human monocytes were isolated using an EasySep CD14^+^ Selection Kit (StemCell Technologies) according to manufacturer’s instructions.

### Cell lines.

The human THP1 monocytic cell line was purchased from ATCC and authenticated by DNA finger printing (Eurofins). The murine lung cancer cell line LLC1 was a gift from Angelica Loskog (Uppsala University) and the mouse breast cancer cell line EO771 was obtained from Maria Ulvmar (Uppsala University). The mouse neuroblastoma cell line 9464D was initially established from a transgenic mouse model on the C57BL/6 background that overexpressed the *TH-MYCN* oncogene and was obtained from Malin Wickström (Karolinska Institute, Stockholm, Sweden). The Ret melanoma cell line was established from a transgenic mouse model developing spontaneous melanoma (16) and was a gift from Viktor Umansky from DKFZ in Heidelberg, Germany. All cell lines were maintained in the IMDM medium (Thermo Fisher Scientific) supplemented with 10% heat-inactivated FBS and 1% penicillin-streptomycin (Thermo Fisher Scientific) and tested for mycoplasma contamination (MycoAlert, Lonza).

### Establishment of the IRAK3-KO mouse model using CRISPR/Cas9.

To study the function of IRAK3 in immunocompetent mice, 4 exons of the *IRAK3* gene (exon 3–6) were deleted using the CRISPR/Cas9 system (Cyagen Biosciences). In brief, gRNAs targeting the forward and reverse strands of the *IRAK3* gene were designed (35) and selected according to the specificity scores (36). Next, mRNA encoding the Cas9 protein was coinjected with the gRNAs into fertilized eggs of the C57BL/6NTac mice. F0 founder pups were screened for gene deletion using PCR followed by sequencing analysis. A mouse with the largest deletion (9839 bp) was bred with a WT mouse to test germline transmission and to generate the F1 animals. Heterozygous mice from the F1 generation were used for breeding to generate homozygous KO mice. Genotyping of the F1 mice was carried out using PCR with the forward primer 1 (5′-TCTTTCGTGAGACACAACACAGAG-3′), forward primer 2 (5′-GTCCCTTTCATAGCCAGTACCAG-3′) and the reverse primer (5′-CGCCTTAAGGTCCTAAAATGTCTT-3′). The homozygous KO genotype shows 1 band at the 466 bp position, while the WT mice show 1 band at the 695 bp position (Supplemental Figure 3A). Homozygous KO mice were viable and did not demonstrate health-related issues within the timeframe of the experiments.

### Syngeneic murine tumor models.

In order to study the effect of *IRAK3* deletion on tumor growth, age-matched (6–12 weeks) female C57BL/6NTac or *IRAK3* homozygous CRISPR-KO mice were s.c. injected with syngeneic cell lines. Mice were checked regularly and tumor volumes were calculated according to the formula (length × width^2^)/2. The maximum tumor volume in the studies was 1,500 mm^3^. In some experiments, mice were treated with ICB antibodies (200 μg αPD-1, clone RMP1-14) or a Rat IgG2a isotype control i.p. in 100 μl PBS 3 times at indicated time points after tumor establishment. In myeloid cell depletion experiments in the Ret melanoma model, WT or *IRAK3-*KO mice were i.p. infused with Rat IgG2a isotype or a CSF-1R depleting antibody (200 μg, clone AFS98) 3 days before tumor implantation and then every 3 days. At the study endpoint, tumors were harvested and cut into small pieces. Single cells were generated using a GentleMacs instrument and a tumor dissociation kit (Miltenyi Biotec). Spleens of the tumor-bearing mice were harvested and crushed through a 40 μm cell strainer, followed by incubating with a red blood cell lysis buffer (Biolegend) on ice for 5 minutes. Single cells were used for flow cytometry analysis on the same day or frozen at –80°C freezers for further analyses.

### Deletion of the human IRAK3 gene using CRISPR/Cas9.

Deletion of the *IRAK3* gene in the THP1 cell line or primary human monocytes was achieved by transfecting cells with the ribonucleoprotein (RNP) complexes containing the *IRAK3* targeting crispr RNAs (crRNAs), trans-activating crispr RNAs (tracrRNAs), and the recombinant Cas9 protein (IDT), using cell-line specific programs on a Neon transfection system (Thermo Fisher Scientific). RNP complexes without the crRNAs were transfected as null controls. For every transfection, 1 μL of *IRAK3*-specific crRNA (100 μM) was annealed to 1 μL tracrRNA (100 μM) in the 1.7 μL IDT duplex buffer by incubating at 95°C for 5 minutes, followed by cooling to 4°C. Next, 1 μL Cas9 protein (2 mg/mL, IDT) was added and incubated for 15 minutes at room temperature to allow RNP complex formation. Prior to transfection, 0.3 μL carrier DNA (100 μM) was added to enhance gene editing efficiency. Primary human monocytes or THP1 cells (4 × 10^6^) were resuspended in 5 μL buffer T or buffer R, respectively, and mixed with equal volume of the RNP complex. After transfection, monocytes were immediately transferred into prewarmed IMDM media supplemented with 10% pooled human AB serum (blood center, Uppsala University Hospital), 100 ng/mL rhGM-CSF (Peprotech) and 1% penicillin-streptomyin (Thermo Fisher Scientific). Cells were incubated for at least 3 days before confirming IRAK3 protein expression and further functional studies.

### Western blotting.

To determine the protein expression, cells were resuspended in the RIPA buffer (Thermo Fisher Scientific) supplemented with the protease/phosphatase inhibitor cocktail (Thermo Fisher Scientific) at 4°C for 15 minutes. Next, samples were centrifuged at 16,000*g* for 15 minutes at 4°C and supernatants were collected followed by storage at –80°C. Protein concentration in the samples were determined with the Bicinchoninic Acid (BCA) Assay (Thermo Fisher Scientific) according to the manufacturer’s instruction. Next, proteins were treated with 6× SDS loading dye and denatured at 70°C for 10 minutes. Equal amount of protein were loaded in the 4–12% Bis-Tris precasted gels (Invitrogen) for SDS-PAGE, then transferred onto nitrocellulose membrane with iBlot Transfer System (Invitrogen). The membrane was blocked with 5% skimmed milk blocking solution followed by incubation with primary antibodies against the target proteins or housekeeping proteins overnight at 4°C. Next, membranes were incubated in corresponding HRP-conjugated secondary antibodies for 1 hour at room temperature on a rocking shaker, followed by visualization using substrate solution in an Amersham Imaging System (GE Healthcare). Densitometric analysis of protein bands was performed using Image Lab 6.1 software (Bio-Rad).

### Cytokine analysis.

Control or IRAK3–KO human primary monocytes or THP1 cells were harvested and seeded in a U-bottom 96-well plate at 0.25 × 10^6^ cells per mL in 200 μL media per well. TLR agonists Pam3CSK4 (TLR1/2), LPS (TLR4) and R848 (TLR7/8) were added to the wells in a dose-dependent manner as indicated in Figure 4D. PBS was added in the control wells. In experiments where MAPK or ERK inhibitors were used, cells were first incubated with 1 μM compound or DMSO control for 2 hours, followed by the addition of TLR agonists. Cells were incubated for 5 hours and supernatants were harvested for cytokine analysis using a 13-plex human antivirus Legendplex Kit (Biolegend) or an Olink inflammation panel that detected 92 soluble factors. To test the effects of STING agonist (ADU-S100), THP1 control or IRAK3-KO cells were seeded with the compound at indicated concentrations and supernatants were harvested after 24 hours for cytokine analysis.

To study response to TLR activation in mouse macrophages, bone marrow cells isolated from the WT or *IRAK3-*KO mice were differentiated in 100 ng/mL rmGM-CSF (Invitrogen) for 4 days in a 6-well plate and seeded at 5 × 10^4^ cells/mL in 200 μL media in a 96 well U-bottom plate. TLR agonists were added and supernatants were harvested after 5 hours for cytokine analysis using a 13-plex Mouse Macrophage/Microglia Legendplex Kit (Biolegend).

### Quantification of mRNA expression.

mRNA contents were isolated from cells using the RNAeasy Mini Kit (Qiagen) and DNA contamination was removed using DNAse I (Thermo Fisher Scientific) and the RNA Clean and Concentrator Kit (Zymo Research). The purity of mRNA samples was confirmed using conventional PCR and visualized using SYBR safe staining (Invitrogen). Primer pairs for the mouse *IRAK3* gene were designed according to exon 3, 4, and 5 in the KO region, and β-actin was used as a reference gene. Next, mRNA expression was quantified using the SsoAdvanced Universal SYBR Green Supermix (Bio-Rad) on a CFX96 Real-Time System (Bio-Rad) and StepOnePlus Real-Time PCR System instruments. Data were analyzed with the CFX Maestro (Bio-Rad) and StepOne (Thermo Fisher Scientific) Software. Changes in gene expression were calculated with 2^–ΔΔCT^ formula and normalized according to data from control samples. To determine the expression of a larger panel of genes in mouse bone marrow cells after LPS treatment, mRNA samples were prepared and analyzed using the nCounter Myeloid Innate Immunity Panel (Nanostring). Normalized mRNA counts can be found in Supplemental data 1.

### Proteomics and phospho-proteomics.

To elucidate the role of IRAK3 on protein expression and pathway activation, global proteomics and phospho-proteomics were performed using LPS-treated primary human monocytes by the proteomics core facility at Uppsala University using an established protocol. In brief, monocytes were isolated from 4 healthy blood donors and cells were transfected with null RNP complex or *IRAK3*-targeting RNP complex as described above. Cells were harvested and seeded at 1 × 10^6^ per mL in 3 mL medium in a 6 well plate, followed by treatment with 1 μg/mL LPS or equal volume of PBS. After 45 minutes, cells were harvested and washed, then stored at –80°C.

Global protein expression and phosphorylation were quantified at the Mass Spectrometry Based Proteomics Facility at Uppsala University. In brief, cell pellets were lysed in 1% β-octyl glucopyranoside and 6M urea containing lysis buffer using a sonication probe and centrifuged at 14,000*g* for 10 minutes. Supernatants were harvested and quantified using a standard DC protein assay. Protein extracts were reduced, alkylated, and on-filter digested by trypsin. For phospho-proteomics analysis, the dimethyl labelling approach was applied. Phosphorylated peptides were enriched using the TiO2 beads and eluted in 1% ammonium hydroxide solution and purified by the Pierce C18 Spin columns (Thermo Fisher Scientific). The peptides were separated in reversed-phase on a C18 column with 150-minute gradient and electrosprayed on-line to a Q-Exactive Plus mass spectrometer (Thermo Fisher Scientific). The data files were analyzed using the MaxQuant software and annotated. Raw data can be found in Supplemental Data 2 and 3.

Proteomics analysis was conducted for all 4 donors, while 1 donor was omitted for phospho-proteomics analysis due to insufficient protein quantity. Unique proteins were defined as proteins that were identified at least in 3 replicates from 1 group but not identified in any samples from the other group. Only unique proteins and proteins identified in at least 2 paired samples were kept for further analysis.

### Analysis of the global proteome in primary human monocytes.

The difference in protein abundance was quantified by (a) calculating the ratio between KO and WT LFQ intensities, (b) averaging the results across the 4 sample pairs, and (c) calculating the log_2_ fold change (FC). A 2-tailed paired T test was applied to procure statistical significance between the 2 groups, which was later corrected by applying False Discovery Rate (FDR). Next, the threshold for differentially expressed proteins was decided as the mean ± 2 SD. Over representation analyses using clusterProfiler (37, 38) were applied to uncover pathway enrichment using GO Biological Process and Reactome databases. STRING network analysis was performed using the online STRING database (39) with the unique and differentially expressed proteins as input. Visualization of the interaction network and cluster pathway enrichment was performed using Cytoscape version 3.9.0 (40).

### Validation of protein phosphorylation.

Control or IRAK3-KO THP1 cells or primary human monocytes were treated with PBS or 1 μg/mL LPS in a 6 well plate at 37°C. Cells were harvested after 45 minutes and cell lysates were prepared using RIPA buffer supplemented with protease/phosphatase inhibitors. Phosphorylation of 39 kinases was detected simultaneously using a Proteome Profiler Phospho-Kinases Kit (R&D systems). To quantify the phosphorylation of individual proteins, control or IRAK3-KO THP1 cells were seeded at 5 × 10^5^ cells per mL in 200 μL medium in a 96 well V-bottom plate, followed by activation with TLR agonists in a dose-dependent manner. The wells without TLR agonists were used as controls. Cells were incubated at 37°C and cell lysates were generated using lysis buffer 6 (R&D systems). Phosphorylation of CREB (S133) or HSP27 (S78/82) was quantified using Duoset IC ELISA kits (R&D systems), and optic densities at 450 and 560 nm were measured using a BioTek Synergy HTX machine.

### Flow cytometry.

For staining of surface proteins, cells were seeded in a 96 well v-bottom plate and incubated with fixable live/dead cell marker (1:200, Invitrogen) and FcR blocker (1:100, Biolegend) in 20 μL PBS. Cells were washed with PBS and stained in 20 μL of a mastermix solution containing desired antibodies for 20 minutes at 4°C. For staining of intracellular IRAK3 in THP1 cells, cells were incubated in 100 μL BD cytofix/cytoperm buffer (BD Bioscience) for 30 minutes at 4°C and cells were washed with 1× perm/wash buffer (BD Biosciences). Rabbit-anti-human IRAK3 antibody (Atlas Antibody) or the rabbit-IgG control (R&D systems) were labeled with a Zenon rabbit IgG fluorescence labeling kit (Invitrogen), according to the manufacturer’s protocol. Cells were then incubated with 0.1 μg fluorescence-labeled antibody or the IgG control in 20 μL PBS for 45 minutes at 4°C. To detect intracellular transcriptional factors (TCF1 and FOXP3) and cytokines (IFNG and TNFA), cells were fixed and permeabilized using a FoxP3 Staining Buffer Set (eBioscience) for 30 minutes at 4°C, followed by washing and incubation with respective antibodies. All cells were resuspended in 200 μL PBS and the results were recorded on a BD Fortessa or a Cytoflex flow cytometer.

### T cell proliferation assay.

Human naive CD3^+^ cell isolation from CD14^+^ cell depleted–PBMC provided by healthy donors was performed with the EasySep CD3^+^ selection kit (Stemcell) according to the manufacturer’s instructions. CD3^+^ cells were resuspended with 1X PBS and labelled with 1.5 μM Cell Trace Violet (CTV) (Invitrogen). Following centrifugation at 500*g* for 5 minutes, cells were resuspended in IMDM medium supplemented with 10% FBS and stimulated with 2 μL per mL of Human CD3/CD28 T Cell Activator (Stemcell). CTV-labelled human CD3^+^ cells were seeded into U-bottom 96-well plate at 50 × 10^3^ cells/100 μL. Control or IRAK3-KO THP-1 cells were harvested and resuspended in IMDM medium supplemented with 10% FBS at 500 × 10^3^ cells/mL. These cells were cocultured with CTV-labeled human CD3^+^ cells at the THP-1 cell-to-CD3^+^ cell ratios of 1 to 1, 0.5 to 1, 0.25 to 1, 0.125 to 1, and 0.0625 to 1, where 1 equals 5 × 10^4^ cells, for 96 hours. Nonstimulated CD3^+^ cells and CD3^+^ cells stimulated with Human CD3/CD28 T Cell Activator without THP-1 coculture were used as control. Alternatively, fresh monocytes were isolated from PBMC using CD14^+^ positive selection (Stemcell). Control or IRAK3 KO human monocytes were cocultured with 5 × 10^4^ allogeneic CD3^+^ T cells at the ratios indicated in the figure legend in IMDM medium supplemented with 10% pooled human AB serum. Cells were harvested following incubation for 5 days. For FACS analysis, cells were collected and labelled with live/dead marker (Invitrogen) and, CD3, CD4, and CD8 T cell markers (Biolegend). Proliferation analysis of CD3^+^CD4^+^ and CD3^+^CD8^+^ T cells was performed with Cytoflex device and FlowJo software (BD Biosciences) according to changes in fluorescence intensity of CTV dye.

### Analysis of RNA-Seq data from patients with bladder cancer.

All data from our analysis can be found in Supplemental data 4.

Transcriptome patient data was obtained from the IMvigor210 trial, which comprised 348 samples. The study originally evaluated the efficacy and safety of atezolizumab, a PD-L1 blocking antibody, in patients with locally advanced or metastatic urothelial carcinoma (13). Bulk RNA-Seq raw counts and clinical data were obtained from http://research-pub.gene.com/IMvigor210CoreBiologies/ After excluding those without response data, a total of 298 patients were included in this study. The raw count data were normalized using the variance stabilizing transformation from DESeq2 (41). Patients below the 25% quantile of *PTPRC* expression were characterized as poorly infiltrated and excluded from any further analysis, leaving the final data set with 212 samples. To calculate IRAK3 levels without skewing for the amount of immune infiltration, the ratio of *IRAK3*-to-*PTPRC* was provided for normalization. Patients above the 75% quantile were considered IRAK3^hi^ and those below the 25% quantile were considered IRAK3^lo^ in all further experiments. A Cox proportional hazards regression model was fit to calculate differences in the overall survival among the different groups (*IRAK3^hi^, IRAK3^medium^*, and *IRAK3^lo^*). The Kaplan-Meier estimate was used to plot the survival curves. The odds ratio between *IRAK3^hi^* and *IRAK3^lo^* was also calculated to evaluate the strength of association between IRAK3 levels and response type. To calculate the differences in the mean *IRAK3/PTPRC* levels in the response groups (CRPR, SD and PD), an ANOVA test with Tukey’s correction was applied.

Differential gene expression and pathway analysis were then investigated for patients in the IMvigor210 trial. Only genes with a read count higher than 1 in more than 10% of the samples were considered, resulting in 20,256 genes. The differential gene expression between *IRAK3^hi^* versus *IRAK3^lo^* was calculated using the Wald test from DESeq2. Independent hypothesis testing was applied to further increase statistical power (42).

To assess the prognostic value of *IRAK3* in nonICB-treated patients, clinical and transcriptome data from patients with bladder cancer (*n* = 408) (15) was downloaded from the online cBioPortal database (http://www.cbioportal.org/) and analyzed. Only patients with an AJCC pathologic tumor stage IV (*n* = 176) were kept for further study to provide a better comparison with the IMvigor210 cohort. The cohort was processed using the same normalization and patient selection pipeline as described above to obtain the 3 *IRAK3* score groups, whose survival were subsequently studied by fitting a Cox proportional hazards regression model.

Functional analysis was performed via Gene Set Enrichment Analysis (GSEA). The package GSEABase was used to query the following collections: biological process database from Gene Ontology, KEGG gene sets, Reactome, Hallmark gene sets (43), Biocarta, PID, and WikiPathways. All collections were downloaded from the Molecular Signature Database (44). The normalized enrichment score was used to compare the enriched and depleted pathways among *IRAK3^hi^* and *IRAK3^lo^* patients. GSVA was then applied to corroborate the individual enrichment of the significant pathways found in GSEA (45).

To further evaluate *IRAK3* coexpression with other genes, a Spearman correlation matrix was calculated for *IRAK3* and all the genes with an adjusted *P* value below 0.001. All 223 samples were used in this experiment. Since the interest was to study the change in gene expression and correlate it with changes in the immune cell compartment, all differentially expressed genes were normalized by calculating their ratio with *PTPRC* beforehand. The FDR was computed to correct for multiple testing.

CIBERSORTx was used on the DESeq2-normalized counts to estimate the proportion of immune cell infiltration in the patient samples (16). The matrix LM22 was used as a reference for deconvolution. The following job parameters were applied: B-mode batch correction, disabled quantile normalization, and a hundred permutations. CIBERSORTx was run in absolute mode and the individual results were Z-scored for visualization.

To test for differences in the immune cellular microenvironment between *IRAK3^hi^* and *IRAK3^lo^* patients, a linear regression model was fit. The empirical Bayes moderated t-statistic from limma package (46) was used to calculate the *P* value for each cellular entity, with an adjusted FDR to correct for the multiple comparisons.

### Analysis of scRNA-Seq data.

To investigate *IRAK3* mRNA expression pattern in bladder cancer, single cell RNA-Seq data from a patient with chemo-resistant metastatic, muscle-invasive urothelial bladder cancer was analyzed (14). Data was accessed through the NCBI Gene Expression Omnibus database accession number: GSE145140. Only the cells from the original biopsy were used. The data set was processed and analyzed using Seurat. A total of 2,075 cells were kept after quality control and normalization, which were clustered into 10 main cell types. Visualization of the results was performed using the SCpubr R package (47).

### Statistics.

Raw data from flow cytometry analysis were processed using the FlowJo V10 software. Results were summarized and tested for statistical significance using the GraphPad Prism software using unpaired 2-tailed *t* tests, nonparametric Mann-Whitney U tests, or 2-way ANOVA, as indicated in the figure legends. *P* values lower than 0.05 were considered significant. Patient survival was compared using a Kaplan-Meier curve and fitted with a Cox proportional hazards regression model. Multivariant analysis was performed using the SIMCA software. In some cases, the volcano plots were generated using the VolcaNoseR web tool (48).

### Study approval.

All animals were maintained under germ-free conditions at the facility in the Rudbeck Laboratory at Uppsala University under an approved ethical permit (Dnr: 5.8.18-06394/2020) by the Swedish Board of Agriculture at Jönköping, Sweden. Buffy coats from healthy donors were received from the Uppsala University Hospital. No ethical approval was needed because blood donors were anonymous.

## Author contributions

YM and GT conceived the study and designed the experiments. GT, MRB, DN, PF, and IP conducted the experiments. MRB and YM designed and conducted bioinformatics experiments. GT and MRB analyzed the data in collaboration with YM. All authors contributed to the writing and revision of the manuscript. GT and MRB are co–first authors and YM is the corresponding author. GT is listed as the first co–first author because of his larger contribution on the conceptualization and experimental research in the project. MRB is listed as the second co–first author because of her larger contribution on the analysis of patient and omics data sets.

## Supplementary Material

Supplemental data

Supplemental data set 1

Supplemental data set 2

Supplemental data set 3

Supplemental data set 4

## Version 1 02/09/2023

In-Press Preview

## Version 2 04/03/2023

Electronic publication
