# Supplementary material for: IL-1 receptor–associated kinase-3 acts as an immune checkpoint in myeloid cells to limit cancer immunotherapy
Source: J Clin Invest. 2023 Apr 3;133(7):e161084. doi: 10.1172/JCI161084 (PMC10065081; doi:10.1172/JCI161084)

Supplemental figures and legends

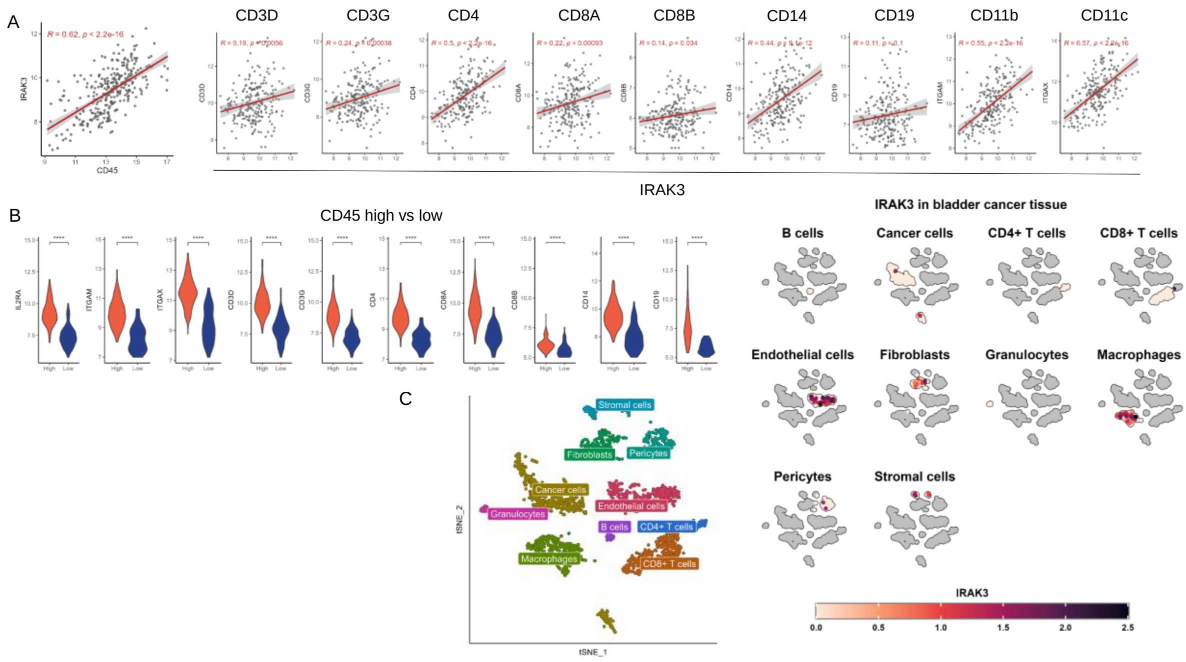

Figure S1. Supplementary figure to Figure 1. A) Correlation between *IRAK3* mRNA expression and lineage-specific genes in the IMvigor210 cohort. **B)** Expression of immune related genes in *CD45* high and low patients. **C)** Expression of *IRAK3* mRNA at the single cell level in bladder tumors using a public scRNAseq dataset.

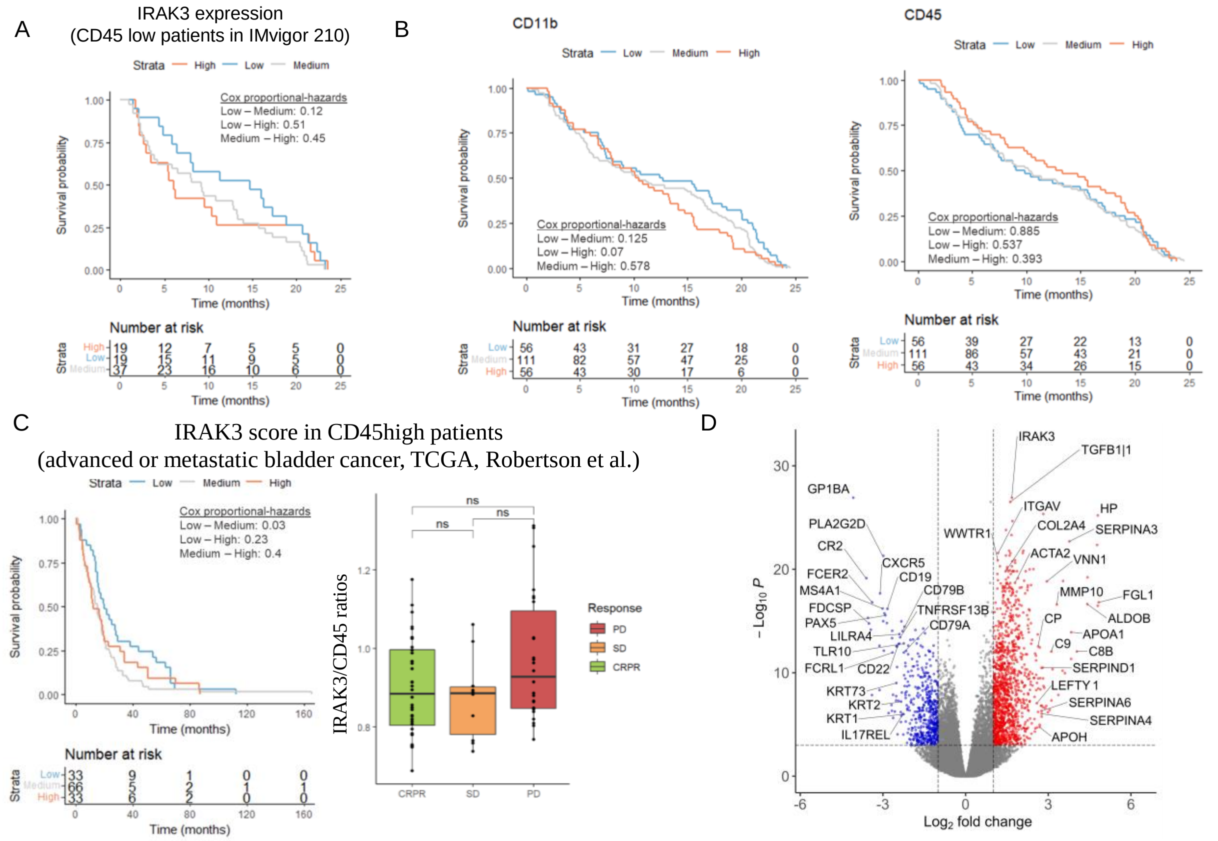

Figure S2. Supplementary figure to Figure 1. A) Patient survival analysis using pre-therapy *IRAK3* mRNA expression in *CD45* low patients of the IMvigor210 cohort. **B)** Patient survival analysis using pre-therapy *CD11b* or *CD45* expression in *CD45* high patients of the IMvigor210 cohort. **C)** Patient survival and response analysis using *IRAK3* mRNA expression in *CD45* high advanced or metastatic bladder cancer patients using a dataset from TCGA. Survival analysis was done using a Kaplan-Meier curve and fitted with a Cox proportional hazards regression model. **D)** Volcano plot to demonstrate up- and down-regulated genes when comparing *IRAK3* high and *IRAK3* low patients in the IMvigor210 trial. Differences were calculated using the Wald test from DESeq2.

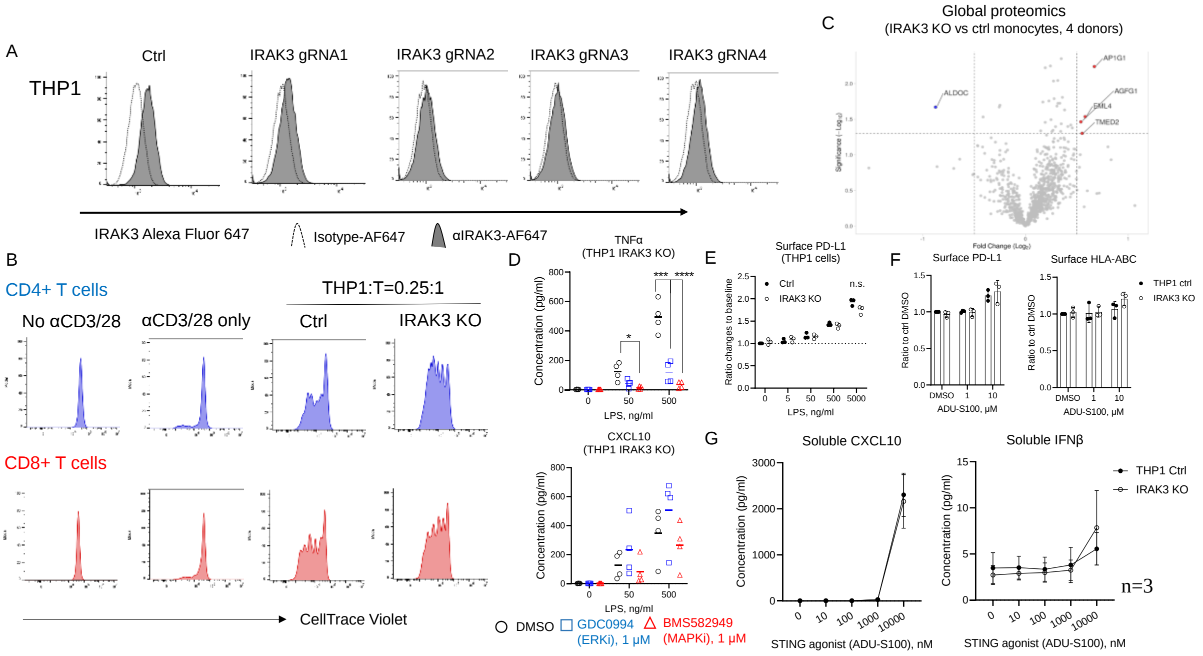

Figure S3. Supplementary figure to Figure 2. A) Comparison of 4 gRNAs targeting the *IRAK3* gene in human THP1 cells. Representative histogram of 3 experiments. **B)** Representative histograms to demonstrate T cell proliferation in the THP1:T cell co-culture assay. **C)** Volcano plot for global protein quantification upon IRAK3 protein deletion in primary human monocytes from 3 individual donors. Log2 fold changes and Log10 P values in KO vs control monocytes for each protein were plotted. **D)** IRAK3 deficient THP1 cells were treated with 1 μM pharmacological inhibitor against MAPK (BMS582949) or ERK (GDC0994) or DMSO for 2 hours, followed by activation using LPS as indicated in the graphs. Supernatants were harvested after 5 hours and cytokines were analyzed using Legendplex (4 biological replicates, unpaired T tests, *:*P*<0.05, ***:*P*<0.001, ****:*P*<0.0001). **E)** Surface PD-L1 expression on control or IRAK3 KO THP1 cells after LPS treatment for 24 hours and normalized to the non-stimulated controls, 3 biological replicates. **F)** Surface expression of PD-L1 or HLA-ABC (n=3, normalized to DMSO controls) or **G)** release of CXCL10, IFNB (n=3) after treatment with a STING agonist, ADU-S100 for 24 hours. Statistical analyses were performed using unpaired T tests in all experiments.

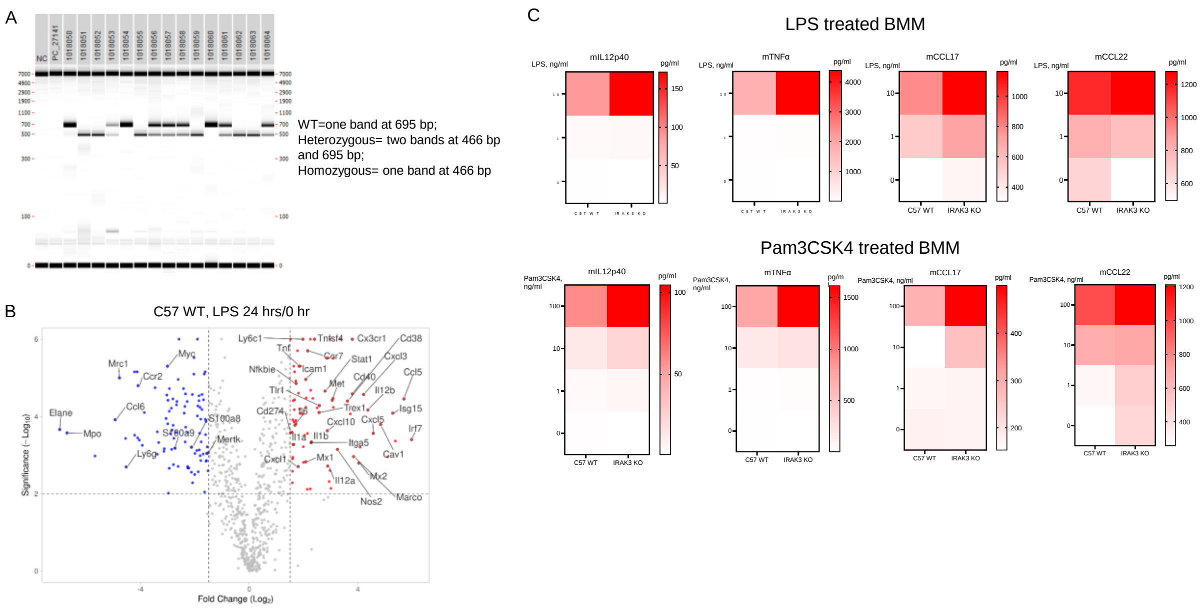

Figure S4. Supplementary figure to Figure 5. A) Genotype results for wild-type (*WT*), heterozygous and homozygous *IRAK3 KO* mice. **B)** Volcano plot to demonstrate differentially expressed mRNAs upon LPS treatment in bone-marrow derived macrophages (BMM) in *WT* mice (n=3) after 24 hours (Log2 fold changes versus Log10 P values, unpaired T tests). **C)** Release of soluble factors from *WT* or *IRAK3 KO* BMM cells in response to LPS or Pam3CSK4 from at least 4 animals in each group.

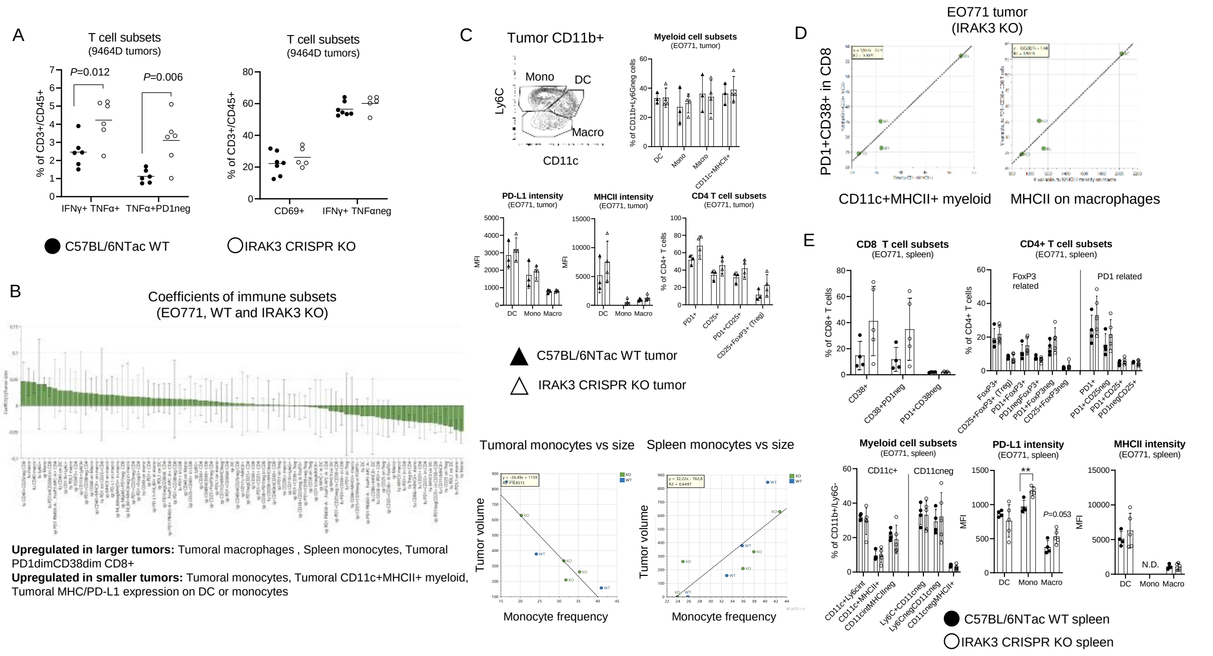

Figure S5. Supplementary figure to Figure 6. A) Percentages of T cell subsets expressing TNFA, IFNG, CD69 or PD-1 were shown in 9464D tumors implanted to *WT* or *IRAK3 KO* mice. Values from individual mouse were shown in the graphs and statistical analysis was done using unpaired T tests. **B)** Multi-variant analysis to identify immunological changes due to tumor volumes using the SIMCA software. **C)** Immunological changes in EO771 tumors from *WT* (n=3) or *IRAK3 KO* mice (n=4). **D)** Correlation between PD-1+CD38+ cytotoxic T cells and myeloid cell activation in EO771 tumors from *IRAK3 KO* mice (n=4). **E)** Immunological changes in spleens from EO771-bearing *WT* (n=4) or *IRAK3 KO* (n=5) mice. Age-matched female C57BL/6NTac or *IRAK3 KO* mice were used, unpaired T tests, **:P<0.01.

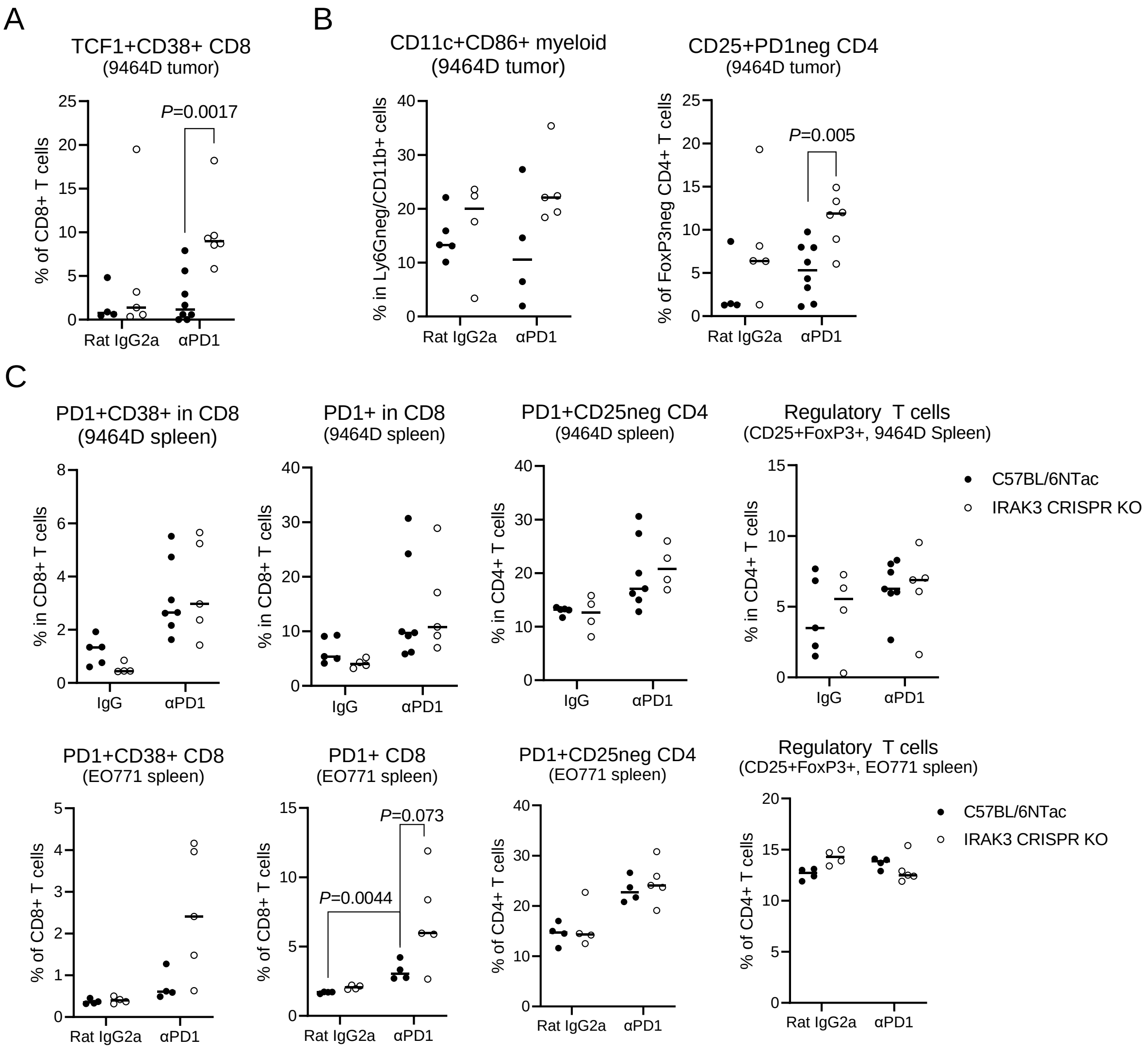

Figure S6. Supplementary figure to Figure 7. A) and **B)** Immunological changes in mice bearing the 9464D tumors from the 4 treatment groups. **C)** Immunological changes in spleens from *WT* or *IRAK3 KO* mice bearing 9464D or EO771 tumors after treatment of Rat IgG2a isotype or an anti-PD-1 antibody. Age-matched female C57BL/6NTac or *IRAK3 KO* mice were used. Number of animals were indicated in the plots. Statistical tests were done using unpaired T tests and P values were shown in the graphs.

Supplementary Table 1: Antibodies

Name	Clone	Application	Product information
Anti-human IRAK3	Rabbit IgG	WB	Cell Signaling Technology/4369
Anti-mouse IRAK3	Goat IgG	WB	Everest/EB08674
Anti-Rabbit IgG HRP-linked Antibody	Goat IgG	WB	Cell Signaling Technology/7074S
Anti-mouse IgG HRP-linked-Antibody	Horse IgG	WB	Cell Signaling Technology/7076S
Anti-human/mouse Vinculin	nVin-1, mouse IgG1	WB	Sigma Aldrich/V9131
Anti-goat IgG (H+L) Secondary Antibody, HRP	Rabbit IgG	WB	Invitrogen/81-1620
Anti-human IRAK3	Rabbit IgG	FACS	Atlas Antibodies/HPA043097
Rabbit polyclonal IgG	Rabbit IgG	FACS	R&D/AB-105-C
APC anti-human CD3	HIT3a, mouse IgG2a,k	FACS	Biolegend/300312
PE anti-human CD4	OKT4, mouse IgG2b,k	FACS	Biolegend/317410
PerCp-Cy5.5 anti-human CD8	RPA-T8, mouse IgG1,k	FACS	Biolegend/301032
PE-Cy7 anti-human CD56	HCD56, mouse IgG1,k	FACS	Biolegend/318318
Brilliant Violet 650 anti-human CD19	HIB19, mouse IgG1,k	FACS	Biolegend/302238
APC-Cy7 anti-human CD25	BC96, mouse IgG1,k	FACS	Biolegend/302614
PE-Dazzle anti-human CXCR3	G025H7, mouse IgG1, k	FACS	Biolegend/353736
FITC anti-human PD-L1	MIH2, mouse IgG1, k	FACS	Biolegend/393606
APC anti-human HLA-ABC	W6/32, mouse IgG2a, k	FACS	Biolegend/311410
PE-Cy7 anti-mouse CD206	C068C2, rat IgG2a, k	FACS	Biolegend/141720
Brilliant violet 650 anti-mouse I-A/I-E	M5/114.15.2, rat IgG2b, k	FACS	Biolegend/107641
PE-Dazzle 594 anti-mouse/human CD11b	M1/70, rat IgG2b, k	FACS	Biolegend/101256
APC-Cy7 anti-mouse Ly6G	1A8, rat IgG2a, k	FACS	Biolegend/127624
PerCp-Cy5.5 anti-mouse Ly6C	HK1.4, rat IgG2c, k	FACS	Biolegend/128012
PE anti-mouse CD11c	N418, Armenian hamster IgG	FACS	eBioscience/12-0114-83
Brilliant violet 421 anti-mouse PD-L1	10F.9G2, rat IgG2b, k	FACS	Biolegend/124315
Brilliant violet 605 anti-mouse CD86	GL-1, rat IgG2a, k	FACS	Biolegend/105037
FITC anti-mouse CD73	TY/11.8, rat IgG1, k	FACS	Biolegend/127219
APC anti-mouse CSF-1R	AFS98, rat IgG2a, k	FACS	Biolegend/135509
APC anti-mouse F4/80	BM8, rat IgG2a, k	FACS	Biolegend/123116
APC-Cy5.5 anti-mouse CD45.2	104, mouse IgG2a, k	FACS	ProSci/155-98-933-0.1
PE anti-mouse CD4	RM4-5, rat IgG2a, k	FACS	Biolegend/100512
PE-Cy7 anti-mouse CD25	PC61, rat IgG1, λ	FACS	Biolegend/102016
APC-Cy7 anti-mouse CD38	90, rat IgG2a, k	FACS	Biolegend/102728
Brilliant violet 421 anti-mouse PD1	29F.1A12, rat IgG2a, k	FACS	Biolegend/135221
PE-Dazzle 594 anti-mouse CD39	Duha59, rat IgG2a, k	FACS	Biolegend/143811
Brilliant violet 605 anti-mouse NKp46	29A1.4, rat IgG2a, k	FACS	Biolegend/137619
PerCP-Cy5.5 anti-mouse CD3	17A2, rat IgG2b, k	FACS	Biolegend/100218
APC anti-mouse FoxP3	FJK-16s, rat IgG2a k	FACS	eBioscience/17-5773-82
PE anti-mouse TCF1	C63D9/rabbit IgG	FACS	Cell Signaling/14456S
FITC anti-mouse IFNG	XMG1.2, rat IgG1, k	FACS	eBioscience/11-7311-81
PE anti-mouse TNFA	MP6-XT22, rat IgG1, k	FACS	eBioscience/12-7321-81
Alexa Fluor 700 anti-mouse CD107a	1D4B, rat IgG2a, k	FACS	Biolegend/121627

PE-Dazzle 594 anti-mouse CD69	H1.2F3, Hamster IgG	FACS	Biolegend/104535
ImmunoCult Human CD3/CD28 T Cell Activator	Antibody complex	In vitro stim	Stemcell/10971
Anti-mouse PD-1	RMP1-14, rat IgG2a, k	In vivo block	BioXcell/BE0146
Rat IgG2a isotype	2A3, rat IgG2a k	In vivo block	BioXcell/BE0089
Anti-mouse CSF-1R	AFS98, rat IgG2a, k	In vivo depletion	BioXcell/BE0213

Supplementary Table 2: other reagents

Name	Application	Product information
Lymphoprep	Cell isolation	StemCell/07851-07861
SepMate tubes	Cell isolation	StemCell/85450
CD14+ positive selection kit	Cell isolation	StemCell/17858
CD3+ positive selection kit	Cell isolation	StemCell/17851
Red blood cell lysis buffer	Cell isolation	Biolegend/420301
Neon transfection system kit	CRISPR KO	Invitrogen/MPK10025
Alt-R® S.p. Cas9 Nuclease V3	CRISPR KO	IDT/1081058
TracrRNA	CRISPR KO	IDT/1072534
ID TE buffer	CRISPR KO	IDT/11-01-02-02
Nuclease free duplex buffer	CRISPR KO	IDT/11-01-03-01
Aqua fixable live/dead marker	FACS	Invitrogen by Thermo Fisher Scientific/L34966A
CellTrace violet	FACS	Invitrogen by Thermo Fisher Scientific/C34557
Zenon dye	FACS	Invitrogen/Z25408
FoxP3 staining buffer set	FACS	eBioscience/00-5523-00
Fixation/Permeabilization Kit	FACS	BD Boisciences/554714
DNase I	Tissue digest	Thermo Scientific/90083
Collagenase IV	Tissue digest	Abnova/P5275.100 MG
Tumor dissociation kit, mouse	Tissue digest	Miltenyi Biotech/130-096-730
GentleMacs C-tubes	Tissue digest	Miltenyi Biotech/130-093-237
MACS smartstrainers	Tissue digest	Miltenyi Biotech/130-110-916
IMDM medium	Cell culture	Gibco/12440-053
Heat inactivated FBS	Cell culture	Gibco/10500-064
Penicillin-streptomycin	Cell culture	Gibco/15140-122
PBS	Cell culture	Gibco /20012-027
MycoAlert mycoplasma detection kit	Cell culture	Lonza/LT07-218
Proteome profiler human phosphor-kinase array	Phospho proteins	R&D systems/ARY003C
Lysis buffer 6	Phospho proteins	R&D systems/895561
pCREB (S133) Duoset IC ELISA	Phospho proteins	R&D systems/DYC2510
pHSP27 (S78/82) Duoset IC ELISA	Phospho proteins	R&D systems/DYC2314
WB gels (NuPAGE 4-12% Bis-Tris Gel)	WB	Invitrogen by Thermo Fisher Scientific/NP0321BOX
Protein Ladder	WB	Thermo Scientific/26619
iBlot 2NC Regular Stacks	WB	Invitrogen by Thermo Fisher Scientific/IB23001
MOPS SDS Running Buffer (20x)	WB	Novex by life technologies/NP0001
Transfer Buffer (20x)	WB	Novex by life technologies/NP0006-1
LDL Sample buffer (4x)	WB	Novex by life technologies/B0007
Skim milk powder	WB	OXOID/LP0033
Ponceaus S solution for electrophoresis (0.2%)	WB	Serva/33427.01
SuperSignal West Pico substrate	WB	Thermo Scientific/34580
SuperSignal West Femto substrate	WB	Thermo Scientific/34095
RIPA Buffer	WB	Thermo Scientific/89900
BCA Protein Assay Kit	WB	Thermo Scientific/23225
Protease and Phosphatase Inhibitor Cocktail	WB	Thermo Scientific/78446
rhGM-CSF	Functional	Peprotech/300-03
rmGM-CSF	Functional	Invitrogen/RP-8620
LPS	Functional	Novus Biologicals/NBP2-25295
R848	Functional	Adipogen Life Sciences/AG-CR1-3582
Pam3CSK4	Functional	Tocris/4633
ADU-S100 ammonium salt STING agonist	Functional	MedChemExpress/HY-12885B
Ravoxertinib-GDC-0994 (ERKi)	Functional	Selleck Chemicals/S7554-5MG

BMS-582949 (MAPKi)	Functional	Selleck Chemicals/S8124-1MG
DNase I	qPCR	Thermo Scientific/EN0521
RNAeasy Mini Kit	qPCR	Qiagen/74104
RNA Clean and Concentrator Kit	qPCR	Zymo Research/R1018
SsoAdvanced Universal SYBR Green Supermix	qPCR	Bio-Rad/1725271
SYBR Safe	qPCR	Invitrogen/S33102

Supplementary Table 3: sequences

Name	Application	Sequence
Hu IRAK3 crRNA1	CRISPR	*CTCCCTTGGCACATTCGAAT*
Hu IRAK3 crRNA2	CRISPR	*AACATTATCCACGGTGACAT*
Hu IRAK3 crRNA3	CRISPR	*TCACCCAAACATACTAGAGT*
Hu IRAK3 crRNA4	CRISPR	*CAGAGCTCTCCGAGCAGCGC*
Mo IRAK3 gRNA1-Reverse	CRISPR	*GGTTGTGAGACCGGACTCCCTGG*
Mo IRAK3 gRNA2-Forward	CRISPR	*CAGTTGAGCTCGATTAGCCCTGG*
Carrier DNA	CRISPR	*CCAGCAGAACACCCCCATCGGCGACGGCCCCGTG CTGCTGCCCGACAACCACTACCTGAGCACCCAGTC CGCCCTGAGCAAAGACCCCAACGAGA*
Mouse IRAK3 Forward Primer	qPCR	*AGCATGCGTGCAGAGAAAAC*
Mouse IRAK3 Reverse Primer	qPCR	*CTCTGGAAGCTGATAGGGGT*
Mouse β-actin Forward Primer	qPCR	*ATGACGATATCGCTGCGCTGGT*
Mouse β-actin Reverse Primer	qPCR	*CCTCGTCACCCACATAGGAGTC*

Supplementary data

Supplementary data 1. Normalized mRNA counts for the mouse bone-marrow derived cells from 3 age-matched female *WT* and *IRAK3 KO* mice with or without LPS stimulation. Related to Figure 5C, 5D and Figure S4B.

Supplementary data 2. Quantification of proteome in control or IRAK3 KO primary human monocytes after 45 minutes LPS stimulation from 4 donors. Data related to Figure 2F, 2G and S3C.

Supplementary data 3. Detection of phosphorylated peptides in control or IRAK3 KO primary human monocytes after 45 minutes LPS stimulation from 3 donors. Data related to Figure 3A.

Supplementary data 4. Analysis of the RNAseq data from the IMvigor210 trial. Data related to Figure 1 and S1 and S2.

Uncropped images for western blotting

Tunali et al.

Full unedited gel for Figure 2B

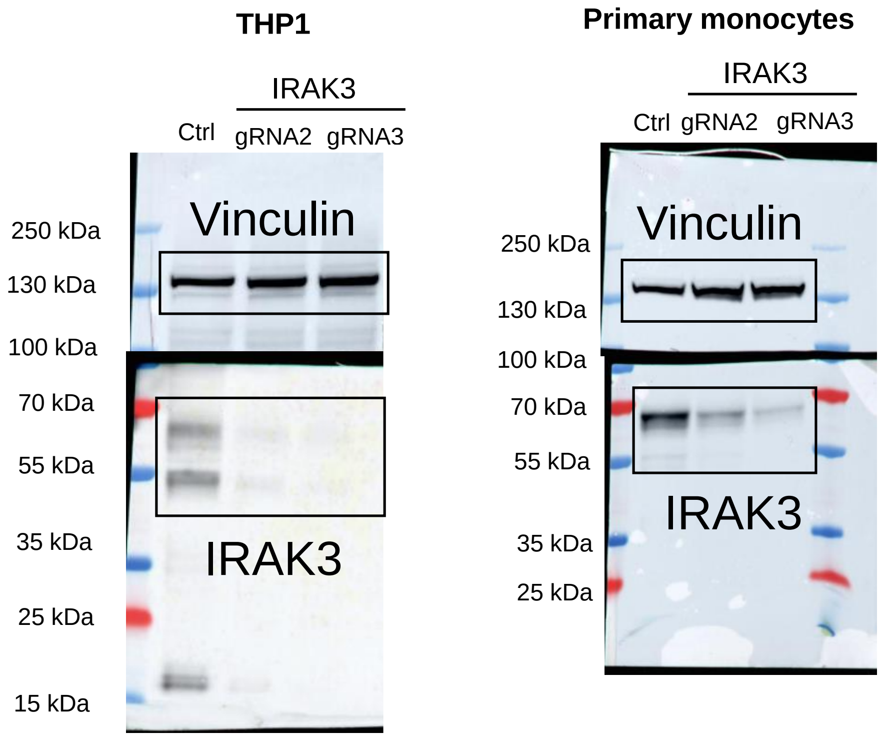

Full unedited gel for Figure 2C

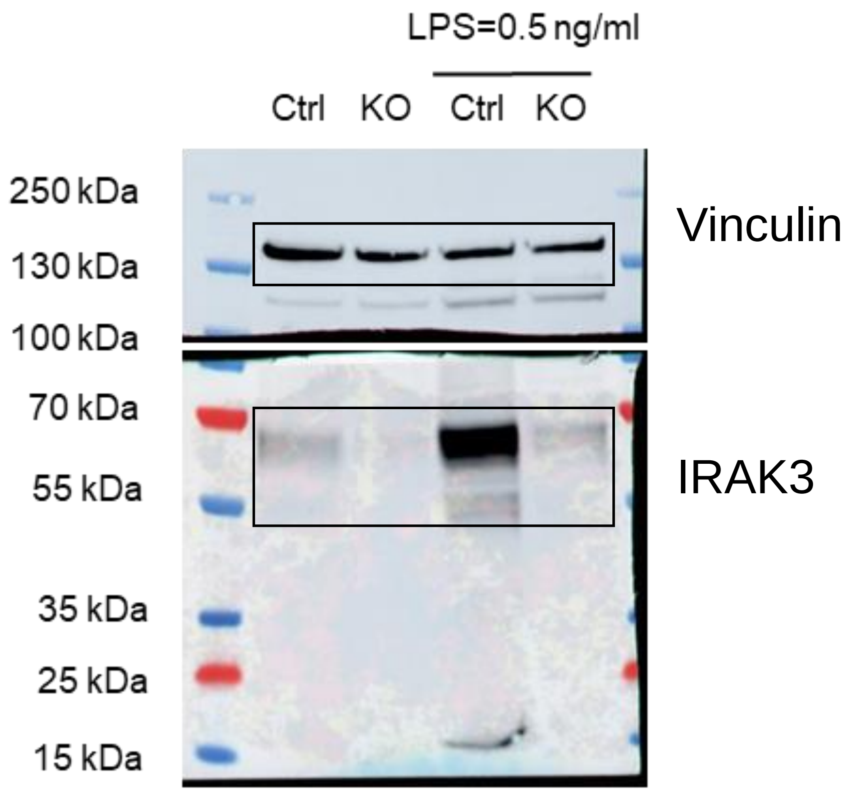

Full unedited gel for Figure 5B

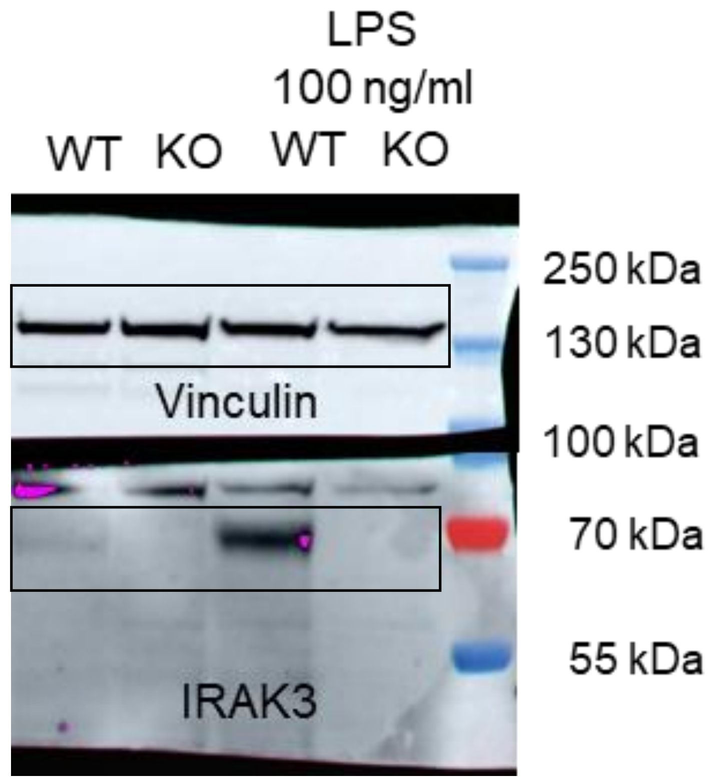

Supplement: Supplemental data [file jci-133-161084-s070.pdf]
